# Genetic modifiers of long‐term survival in sickle cell anemia

**DOI:** 10.1002/ctm2.152

**Published:** 2020-08-12

**Authors:** Ambroise Wonkam, Emile R. Chimusa, Khuthala Mnika, Gift Dineo Pule, Valentina Josiane Ngo Bitoungui, Nicola Mulder, Daniel Shriner, Charles N. Rotimi, Adebowale Adeyemo

**Affiliations:** ^1^ Division of Human Genetics, Institute of Infectious Disease and Molecular Medicine (IDM), Faculty of Health Sciences University of Cape Town Cape Town South Africa; ^2^ Institute of Infectious Disease and Molecular Medicine (IDM), Faculty of Health Sciences University of Cape Town Cape Town South Africa; ^3^ Computational Biology Division, Department of Integrative Biomedical Sciences, Institute for Infectious Disease and Molecular Medicine University of Cape Town Cape Town South Africa; ^4^ Center for Research on Genomics and Global Health National Human Genome Research Institute National Institutes of Health Bethesda Maryland USA

**Keywords:** Africa, genetic modifiers, sickle cell disease, whole exome sequencing

## Abstract

**Background:**

Sickle cell anemia (SCA) is a clinically heterogeneous, monogenic disorder. Medical care has less‐than‐optimal impact on clinical outcomes in SCA in Africa due to several factors, including patient accessibility, poor access to resources, and non‐availability of specific effective interventions for SCA.

**Methods:**

Against this background, we investigated 192 African participants who underwent whole exome sequencing. Participants included 105 SCA patients spanning variable clinical expression: a “long survivor” group (age over 40 years), a “stroke” group (at least one episode of overt stroke), and a “random” group (patients younger than 40 years without overt cerebrovascular disease). Fifty‐eight ethnically matched homozygous hemoglobin A controls were also studied. Findings were validated in an independently recruited sample of 29 SCA patients. Statistical significance of the mutational burden of deleterious and loss‐of‐function variants per gene against a null model was estimated for each group, and gene‐set association tests were conducted to test differences between groups.

**Results:**

In the “long survivor” group, deleterious/loss‐of‐function variants were enriched in genes including *CLCN6* (a voltage‐dependent chloride channel for which rare deleterious variants have been associated with lower blood pressure) and *OGHDL* (important in arginine metabolism, which is a therapeutic target in SCA). In the “stroke” group, significant genes implicated were associated with increased activity of the blood coagulation cascade and increased complement activation, for example, *SERPINC1*, which encodes antithrombin. Oxidative stress and glutamate biosynthesis pathways were enriched in “long survivors” group. Published transcriptomic evidence provides functional support for the role of the identified pathways.

**Conclusions:**

This study provides new gene sets that contribute to variability in clinical expression of SCA. Identified genes and pathways suggest new avenues for other interventions.

## Graphical abstract

This study provides new insights into the pathophysiology of sickle cell anemia (SCA) by identifying gene sets with deleterious/loss‐of‐function variants that contribute to variability in SCA clinical expression. In the “long survivor” group (age over 40 years), enriched genes included CLCN6 (a voltage‐dependent chloride channel for which rare deleterious variants have been associated with lower blood pressure) and OGHDL (important in arginine metabolism, which is a therapeutic target in SCA). In the “stroke” group, enriched genes implicated increased activity of the blood coagulation cascade and increased complement activation; Notable among these genes is SERPINC1 that encodes antithrombin. Identified genes and pathways suggest new avenues for other interventions.

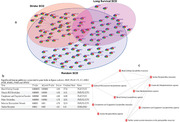

## 1 INTRODUCTION

Sickle cell disease (SCD) is a group of blood disorders caused by mutations in *HBB* that promote polymerization of hemoglobin and sickling of erythrocytes. The most common and most clinically severe form of SCD is sickle cell anemia (SCA [MIM: 603903]) and is caused by homozygosity of the sickle mutation HbS. While the estimated age of the sickle mutation is ∼7300 years,^1^ the mutation has persisted in appreciable frequencies because of the protection that heterozygotes have against malaria. An estimated 305 800 babies are born each year with SCD worldwide, with nearly 75% of the births occurring in sub‐Saharan Africa (SSA).^2^ Despite this high incidence, most SSA countries lack effective public health programs focused on SCD.^3^

Clinical expression of SCD often shows considerable variation in features such as the severity of anemia, the frequency of painful vaso‐occlusive crises, stroke, and mortality. Both genetic and non‐genetic factors are known to influence the severity of SCD. For example, high fetal hemoglobin (HbF) levels have long been known to be associated with less severity^4^; HbF levels are under genetic control and are amenable to therapeutic manipulation.^5^ Similarly, co‐inheritance of α‐thalassemia is protective against some SCD‐related complications, such as hemolysis, stroke, and kidney disease.^6^, ^7^ Availability of appropriate comprehensive medical care for SCD that combines newborn screening, penicillin prophylaxis, screening for stroke risk, transfusion program, and the use of hydroxyurea, in high income countries, usually mitigates morbidity and facilitates longer survival of SCD patients.^8^ This is, however, not the case in most of SSA, due to the combined effect of inappropriate care and often severe clinical complications,^7^, ^9^ compounded by other factors such as malaria, malnutrition, infectious diseases of childhood, and poverty. Thus, up to 90% of infants with SCD in SSA are believed to die needlessly by 5 years of age.^10^

Different *HBB* (MIM: 141900) haplotypes (Cameroon, Central African Republic, Benin, Senegal, and Arabian/Indian) have also been associated with variable clinical expression of SCD.^2^ However, a recent study of sickle *HBB* haplotypes based on sequence data rather than restriction site data led to a reclassification of the classical haplotypes and found sub‐structuring of haplotypes that may have confounded previous associations of the haplotypes with clinical severity.^1^ To search the genome for protein coding genes that affect disease severity, we utilized a whole exome sequence (WES)‐based approach to explore the specific hypothesis that rare gene‐associated and function‐altering variants with potentially high penetrance are associated with SCA clinical sub‐phenotypes,^11^ and could lead to the identification of modifiable pathophysiological pathways. We studied two distinct clinical SCA groups, a “long survivor” group and a “stroke” group, which we contrasted with a “random” group to infer critical genes and pathways. We also contrasted the “long survivor” and “stroke” groups as extreme phenotypes that are likely to come from the relatively benign versus the most severe (respectively) ends of the phenotypic spectrum of SCA.

## 2 MATERIALS AND METHODS

### 2.1 Ethics statement

The study was performed in accordance with the guidelines of the Helsinki Declaration. Ethical approval was given by the National Ethical Committee Ministry of Public Health, Republic of Cameroon (No 033/CNE/DNM/07); and the University of Cape Town, Faculty of Health Sciences Human Research Ethics Committee (HREC RE: 132/2010).

### 2.2 Patients and methods

Recruitment for the discovery group was conducted in Cameroon at the Yaoundé Central Hospital and Laquintinie Hospital in Douala, as previously described.^12^ Socio‐demographic and clinical data including blood counts and hemoglobin (Hb) electrophoresis were obtained on enrolment. The present study was restricted to SCA (ie, HbSS), the most prevalent and severe form of SCD. This study design decision was taken to remove the potential confounding effect of beta‐thalassemia and hemoglobin C, both of which are associated with milder forms of SCD. The discovery sample of patients included three groups. (a) A “long survivor group” comprised SCA patients aged over 40 years; this cut‐off was based on a life expectancy of 43 years for SCA in the Cooperative Study.^4^ (b) A “stroke” group comprised SCA patients with at least one clinical episode of overt stroke, a devastating complication of SCA that is considered to be a proxy of severity^4^ and is influenced by genetic modifiers.^13^ (c) A “random group” comprised patients younger than 40 years with no known cerebrovascular disease and randomly selected from among clinically stable patients. No patient was on hydroxyurea, at the time of recruitment. Fifty‐eight ethnically matched homozygous HbAA controls were randomly selected from apparently healthy blood donors recruited in Yaoundé.^14^ The replication cohort consisted of adult SCA patients (age range 18‐51 years, mean age 26.1 years) from the Demographic Republic of Congo (DRC), recruited at the Haematology Clinic, Groote Schuur Hospital in Cape Town, South Africa.

### 2.3 Sickle cell disease mutation, β‐globin gene cluster haplotypes, and 3.7 kb α‐globin gene deletion

DNA was extracted from peripheral blood. Molecular analysis to determine the presence of the sickle mutation was carried out by polymerase chain reaction (PCR), followed by DdeI restriction analysis.^12^ Using published primers and methods, five restriction fragment length polymorphism (RFLP) sites in the β‐globin gene cluster were amplified to analyze the *HBB* haplotype background.^12^ The 3.7 kb α‐globin gene deletion was screened using expand‐long template PCR.^14^

#### 2.3.1 Whole exome sequencing

DNA samples underwent sequencing at Omega Bioservices, Omega Bio‐tek, Inc, Emory University, USA. The Roche Nimblegen SeqCap EZ MedExome v2.0 (∼47 Mb target), which has enhanced coverage of medically relevant genes, was used for sequence capture. Samples passing quality control, library preparation, and exome capture were sequenced on an Illumina HiSeq 4000 sequencer. Read mapping and alignment were performed as described in the Supporting Information Methods. Because different calling methods produce large numbers of differing variants, we adopted an ensemble approach implemented in VariantMetaCaller^15^ (see Figure S1 and details in Supporting Information Methods).

#### 2.3.2 Variant calling quality control, annotation, and prioritization

Joint variant calling was conducted from three independent callers (Figure S1) on each subject group defined above and across all samples (Supporting Information Methods). Before applying the ensemble approach (VariantMetaCaller^15^) across the resulting variant set from three callers (Supporting Information Methods) from each independent subjects’ group, we filtered each resulting VCF file using the GATK tool Variant Filtration.^16^ Variant filtering procedures and quality control assessment are as described in the Supporting Information Methods. Final call‐sets were produced from VariantMetaCaller.^17^ We used ANNOVAR to perform gene‐based annotation (Supporting Information Methods) on each independent final call‐set per subjects’ group. First, each resulting functional annotated call set was independently filtered for predicted functional status. We used 21 in silico prediction tools (*SIFT, LRT, MutationTaster, MutationAssessor, FATHMM, fathmm‐MKL, RadialSVM, LR, PROVEAN, MetaSVM, MetaLR, CADD, GERP++, DANN, M‐CAP, Eigen, GenoCanyon, Polyphen2 HVAR, Polyphen2 HDIV, PhyloP*, and *SiPhy*) to identify variants whose predicted functional status is “deleterious” (D), “probably damaging” (D), “disease_causing_automatic” (A) or “disease_causing” (D). We retained a variant if it had at least 17 predicted functional status D (Supporting Information Methods). Second, the retained variants from each data set were further filtered for rarity, exonic variants, and nonsynonymous mutations, yielding a final candidate list of in silico predicted mutant variants from each subject group. We also reported the aggregated SiPhy score from all identified SNPs within a gene.

#### 2.3.3 Gene‐specific differences in SNP frequencies

To assess gene set differences in frequencies, we first computed SNP‐specific allele frequencies. Assuming a population evolving under the Wright‐Fisher model under selective neutrality, and with an expected number of mutations, we used a stepwise constant effective population size to (1) compute the allele frequency difference, (2) estimate group pairwise differences, and (3) compute a test statistic for which an excess of large values indicated deviation from the null model (Supporting Information Materials).^18^ Second, assuming SNPs in 40 kb upstream and downstream (exon) within a gene are close and possibly in Linkage Disequilibrium (LD), SNP‐specific unusual allele frequency summary statistics of SNPs in the defined gene region were aggregated as described in Supporting Information Materials to obtain gene‐specific differences in SNP frequencies.

#### 2.3.4 Burden and rare‐variant analyses

Rare variants in a gene or region may influence a phenotype in different directions and with differing magnitude of effect.^11^, ^19^, ^28^ Therefore, gene‐set analyses were performed to determine the associations between genes and phenotype as defined by the three participant categories. Gene sets of variants comprised non‐synonymous, missense, and stop lost/stop variants (in core *in silico* mutant genes) in the exome datasets and in 40 kb upstream and downstream (exon) within a gene were mapped within the coordinates of the gene in the human genome build hg19. We conducted rare variant analysis using the optimal unified sequence kernel association test (SKAT‐O^19^) in the exome dataset for our primary comparisons, namely: (a) Long survivors versus Random SCA (n = 82), (b) Stroke versus Random SCA (*n *= 79), and (c) Stroke versus Long survivors (n = 59). We also analyzed Stroke versus Controls (n = 81), Long survivors versus Controls (n = 84), and Random SCA versus Controls (n = 114). All analyses used an adaptive minor allele frequency threshold of 1/√2*n* (where n is the number of individuals) for definition of “rare variant.”^11^, ^19^ A total number of 17 285 gene sets had two or more rare variants and were included in the SKAT‐O rare variant analysis The SKAT‐O^19^ analysis was performed using a linear weighted kernel with a missingness cutoff of 0.9 and adjustments for covariates including age and principal components of population stratification. To account for multiple testing, empirical *P*‐values were calculated after 100 000 permutations; therefore, a *P*‐value of .05 was considered significant. While permutation testing was used to adjust for multiple testing for each comparison, we also considered the fact that we had three primary hypotheses and adjusting for the number of primary hypotheses led to our considering *P*‐values < .017 (ie, .05/3) as significant.

#### 2.3.5 Network and enrichment analysis

From the gene lists obtained from the analyses of deleterious/loss‐of‐function variants and unusual allele frequency spectra, we next sought to identify networks of interactions using a comprehensive human protein‐protein interaction (PPI) network.^20^ We examined how the genes in these networks were associated with phenotypes, pathways, biological processes, and molecular functions. Pathways and networks were drawn from various bioinformatics databases, including KEGG, Panther, Biocarta, Reactome, and the Gene Ontology (GO) Consortium database (Supporting Information Materials). Enrichment analysis was performed using a custom script as well as the DAVID and PANTHER tools.

## 3 RESULTS

### 3.1 Characterization of participants

Clinical characteristics of the 105 Cameroonian SCA patients are shown in Table 1. The median ages were 44.0, 20.5, and 16.5 years, for the “long survivor,” “stroke,” and “random” SCA groups, respectively. A total of five of 23 (21.7%) patients had stroke before the age of 16 years. “Long survivor” SCA patients had significantly lower leucocyte counts and health care utilization rates. Two *HBB* haplotypes (Benin and Cameroon) were observed in the sample. The overall distribution of haplotypes was not significantly different among the three groups, although it should be noted that a greater proportion of the “stroke” group carried at least one Cameroon haplotype when compared to the other two groups (38.5% vs 9.1% and 15.4%, Table 1). The distribution of the 3.7 kb α‐globin gene deletion genotypes and mean HbF levels did not differ significantly among the three groups.

**TABLE 1:** Characteristics of the 105 SCA patients that underwent WES displayed as median (25th to 75th percentiles) or percent (%)

**Variables**		Random SCA(N = 56)	SCA with overt stroke (N = 23)	Long survivor SCA (N = 26)	*P*‐values^*^
**Age (years)**		16.5 (9.2‐25.7)	20.5 (16.25‐25.75)	44 (41‐49.5)	**<**.**0001**
**Gender**	F/M (50/46)	28/28	12/11	15/12	.854
**Haematological indices **	RBC (10¹²/L)	2.7 (2.2‐3.2)	2.9 (2.4‐3.5)	3.4 (2.6‐3.9)	.101
	Hb (g/dL)	7.8 (7.1‐8.8)	8.2 (7.3‐8.8)	8.2(6.8‐9.8)	.462
	MCV (Fl)	84 (78‐92.5)	84 (73.2‐93.7)	81 (74‐89.5)	.824
	MCHC (g/dL)	33.6 (31.0‐36.6)	34.1 (31.7‐36.5)	32.8 (30.4‐35.1)	.420
	WBC (10^9^/L)	12.4 (10.3‐36.6)	14.4 (10.3‐17.8)	9.4 (8.23‐12.2)	.**026**
	Lymphocytes (10^9^/L)	5.2 (3.9‐6.9)	5.5 (3.5‐7.9)	3.9 (2.7‐4.8)	.**005**
	Monocytes (10^9^/L)	1.3 (1.0‐1.9)	1.3 (0.8‐2.1)	0.9 (0.8‐1.3)	.**022**
	Platelets (10^9^/L)	342.5 (289.2‐342.5)	359.5 (283‐440.5)	329 (228.5‐452.5)	.583
	HbA_2_ (%)	3.7 (3.2‐4.2)	4.0 (3.2‐4.8)	3.6 (3.2‐4.9)	.325
	HbF (%)	13.1 (2.7‐17.3)	10.6 (5.2‐13.7)	9.4 (3‐14.3)	.231
**Clinical events **	Vaso‐occlusive crisis (n/year)	0.6 (1‐5)	2 (1‐3)	2 (1‐3.5)	.172
	Consultations (n/year)	0.5 (0‐5)	1 (0‐6)	0 (0‐0)	.**030**
	Hospitalization (n/year)	0.3 (0.5‐2)	1 (1‐2)	0 (0‐1.5)	.606
	Blood transfusion (%)	75.9	85.0	70.0	.315
	Stroke (%)	0	100	5.0	**<.0001**
**3.7 α‐globin gene deletion genotypes**	αα / αα	57.8%	58.8%	46.7%	.652
	αα/ α3.7	31.1%	29.4%	26.7%	
	α3.7/ α3.7	11.1%	11.8%	26.6%	
***HBB* Haplotype** ^a^	Benin/Benin	90.9%	61.5%	84.6%	.834
	Benin/Cameroon	3.0%	38.5%	7.7%	
	Cameroon/Cameroon	6.1%	0.0%	7.7%	

Abbreviations: RBC, red blood cell counts; Hb, hemoglobin; MCV, mean corpuscular volume; MCHC, mean corpuscular hemoglobin concentration; WBC, white blood cell counts;

a Percentage of individuals not chromosomes.

* *P*‐value in three‐way comparisons; significant *P*‐values are **bolded**.

**FIGURE 1:**
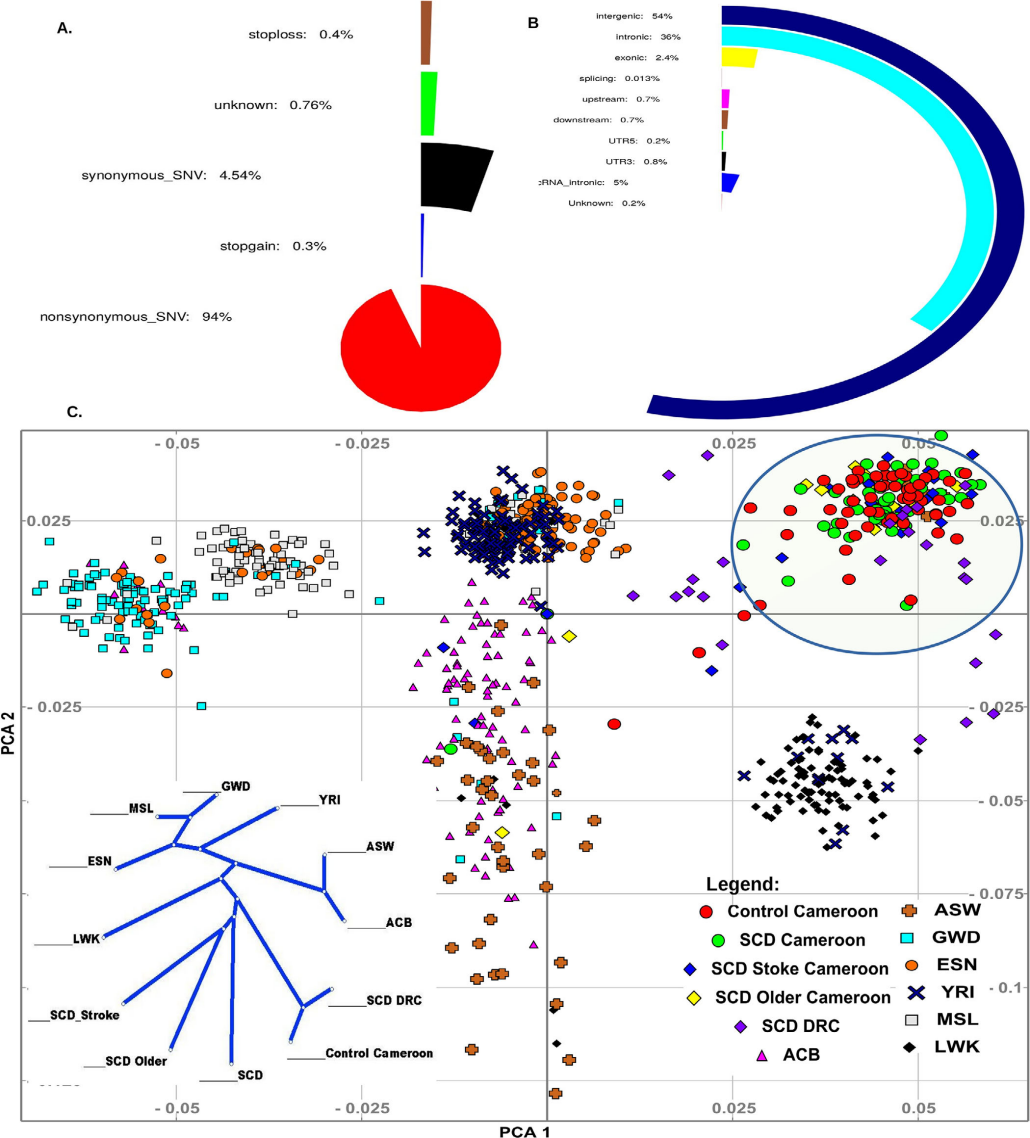
SCA exome map characteristics. A), Percentages of functions covered from 80 226 exonic variants. B, Overall percentage of variant functions from 8 458 386 variants in discovery and replication cohorts. C) Principal component analysis (PCA) plot of the three Cameroonian SCA sub‐groups (“random,” “stroke,” and “long survivor”), and African ancestry samples from 1000 Genomes phase 3 release, indicating the Cameroonian SCD patients and controls are relatively homogeneous and from similar background. Date from DRC participants is scattered in the convex of Western and Eastern African populations, supporting a bantu migration route from west to south Africa^21^

A total number of 8 458 386 variants were called in the whole exome sequence dataset, of which 80 226 were exonic, distributed as 0.4% stop loss, 0.3% stop gain, 4.5% synonymous, and 94% non‐synonymous or splice site variants (Figures 1A,B; Figure S2). Principal component analysis (PCA) of the study sample with the African populations (AFR) from the 1000 Genomes Project showed that the study samples clustered separately from the other Africans (Supporting Information Materials), which is not surprising because samples from Cameroon were not included in the 1000 Genomes Project. PCA plots (Figure 1A; Figure S3) showed no global population differences among the SCA patients and control groups, that is, cases and controls clustered together (Supporting Information Materials). The replication SCA sample from DRC had a median age of 26 years and 13.8% (4/29) had a clinically overt stroke.

#### 3.1.1 Mutational burden of genes in discovery samples

**FIGURE 2:**
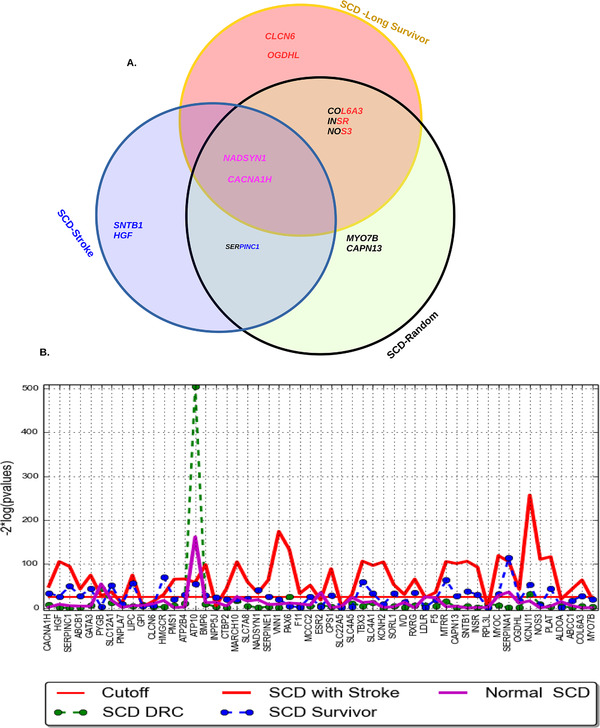
Gene mutations in three Cameroonian SCA sub‐groups (only the 12 genes replicated in patients from Congo are represented here). A, The Venn diagram shows the overlap of the replicated candidate mutations in the three SCA groups. B, Gene‐specific genetic differentiation based on identified 49 mutant genes identified (Table S1), among 58 HbAA Cameroonian controls, each SCA patient sub‐group, and the replication cohort of SCA patients from Congo

**FIGURE 3:**
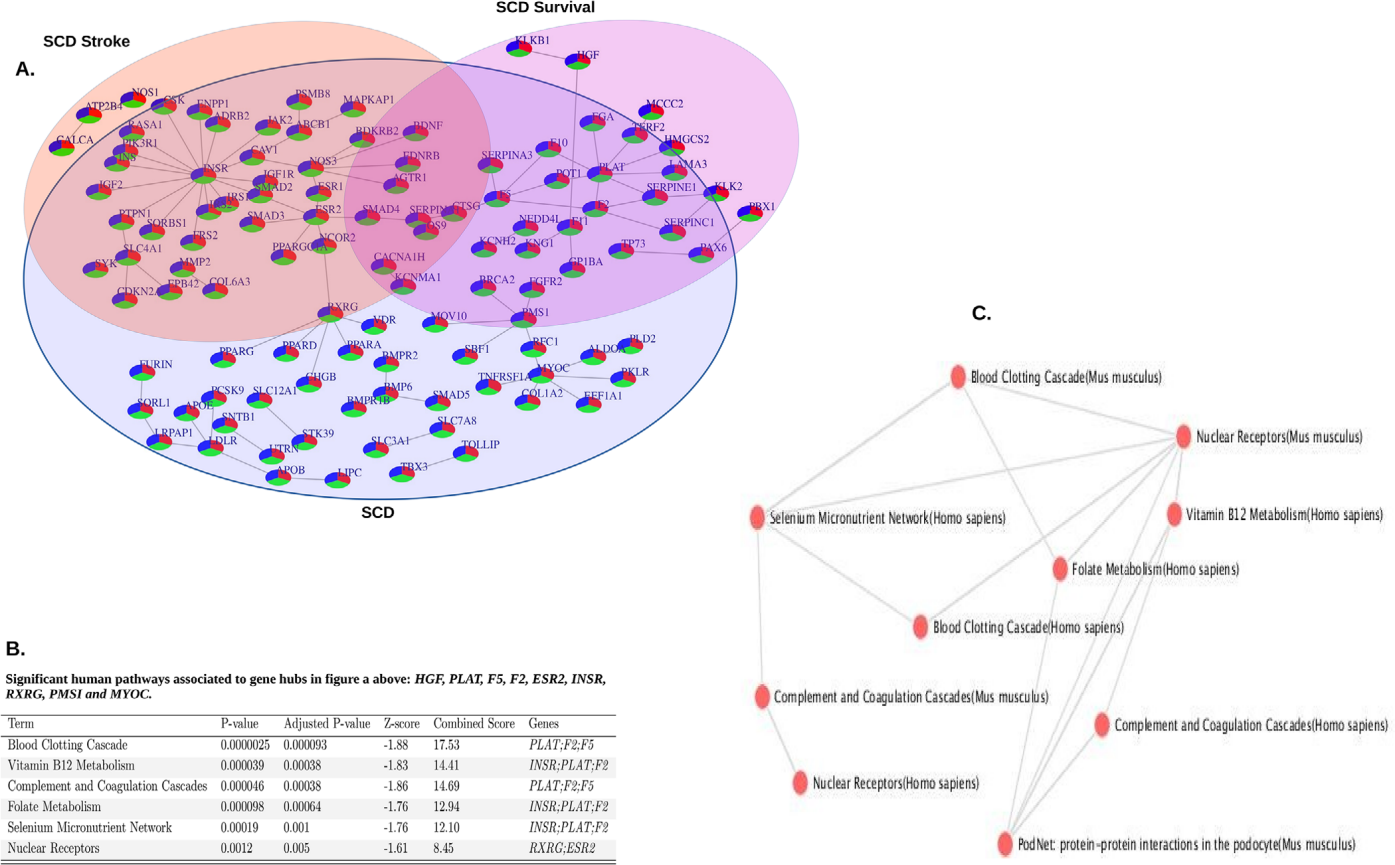
Overlapping biological networks of identified candidate mutations in three Cameroonian SCA sub‐groups. A, Overlap of three networks of the identified candidate mutations (Table S1). B, Significant pathways associated with gene hubs. C, Interaction network of top associated pathways of gene hubs

Among SCA patients in the discovery sample, we detected significant differences in the burden of non‐synonymous, function‐altering variants (Figure 2A) in a total of 49 genes (Table 2; Tables S1 and S5): 17 genes in the “long survivor” group (Figure 3; Figure S4A), 19 genes in the “stroke” group (Figure 3; Figure S5a), and 39 genes in the “random” group (Figure 3; Figure S6A). Five genes were found only in the “long survivor” group (*ATP2B4 [MIM: 108732], CLCN6 [MIM: 602726], OGDHL [MIM: 617513], ESR2 [MIM: 601663]*, and *SLC7A8 [MIM: 604235]*), and a different set of five genes were found only in the “stroke” group (*SLC22A5 [MIM: 603377], HGF [MIM: 142409], IVD [MIM: 607036], ABCC1 [MIM: 158343]*, and *SNTB1 [MIM: 600026]*) (Table S1). No genes overlapped between the “stroke” and “long survivor” groups while also absent from the “random” group (Table S1; Figure 2A). Six genes (*CPS1 [MIM: 608307]*, *PYGB [MIM: 138550]*, *MARCH10 [MIM: 613337]*, *SLC4A5 [MIM: 609802]*, *NADSYN1 [MIM: 608285]*, and *CACNA1H [MIM: 607904]*) were common to all three SCA groups (Table S1). Notably, none of the genes identified among SCA patients (Table S1) were significant in the HbAA control samples.

**TABLE 2:** Genes with high burdens of deleterious and loss‐of‐function mutations in SCA patients in both discovery and replication samples

						*Z*‐scores^¥^				
Gene Max # SNPs^1^	Gene name	cDNA change^£^	Protein change	ExAC AFR	ExAC EUR	“Random” SCA	“Stroke” SCA	“Long survivor” SCA	Replication samples	
*NADSYN1*	12	NAD synthetase 1	c.G175A	p.E59K	0	0	15.15	15.15	15.15	16.21
*CACNA1H*	4	Calcium channel, voltage‐dependent, T type, alpha 1H subunit	c.C1538T	p.S513L	0	0	15.05	15.18	15.05	17.02
*SERPINC1*	5	Serpin peptidase inhibitor, clade C, member 1	c.G973C	p.A325P	0	0	19.75	19.75	–	18.54
*INSR*	3	Insulin receptor	c.G3311A	p.R1104H	0	0	15.83	–	15.83	20.82
*NOS3*	9	Nitric oxide synthase 3	c.G1585A	p.G529S	0	0	15.73	‐	15.73	18.74
*COL6A3*	9	Collagen, type VI, alpha 3	c.T7463C	p.I2488T	0	0	19.472	–	16.95	17.01
*HGF*	1	Hepatocyte growth factor	c.C1595T	p.A532V	0	0	–	19.91	–	18.93
*SNTB1*	4	Syntrophin, beta 1	c.A173G	p.N58S	0	0	–	19.27	–	16.98
*CLCN6*	5	Chloride channel 6	c.G992C	p.C331S	0	0	–	–	19.058	18.84
*OGDHL*	5	Oxoglutarate dehydrogenase‐like	c.C632T	p.T211M	0	0	–	–	19.95	15.97
*CAPN13*	4	Calpain 13	c.C336G	p.I112M	0	0	18.074	–	–	15.76
*MYO7B*	26	Myosin VIIB	c.G77A	p.G26D	0.0006	0	17.660	–	–	20.14

¥ The *z*‐scores are obtained from aggregating the SiPhy (29‐way) score based on identified mutants SNPs within genes (See details in Table S5 of all the mutations found).

£ Exonic. nonsynonymous variants that were considered damaging according to 21 different functional scores from the annotation databases, including SIFT, LRT, MutationTaster, MutationAssessor, FATHMM, fathmm‐MKL, RadialSVM, LR, PROVEAN, MetaSVM, MetaLR, CADD, GERP++, DANN, M‐CAP, Eigen, GenoCanyon, Polyphen2 HVAR, Polyphen2 HDIV, PhyloP, and SiPhy, as previously reported.^8^ Abbreviations: Max #SNPs: Maximum number of nonsynonymous variants observed among the three SCD groups; SNP: Single Nucleotide Polymorphism; ExAC: Exome Aggregation Consortium; AFR: African; EUR: European. The reported cDNA change, Protein change, and all ExAc frequencies are from the putative deleterious variants with top SiPhy (29‐way) score.

Among key candidate genes, with known role in SCA‐related pathophysiology, only *NADSYN1*, which is involved in glutamine pathways, has significantly common mutations in all three subgroups (Table S1). While *SERPINE1* and *SERPINA1* involved in coagulation pathways, had recurrent mutations in the random group only, but not in the two “extreme” groups. However, gene‐set rare‐variant association analyses showed significance difference in *NADSYN1* gene between “Stroke” versus “Random” SCA (Table 3).

**TABLE 3:** Significant genes from gene‐set rare‐variant association analyses

Region	Gene	Min. #Rare Variants tested	“Stroke”Versus“Random” SCA	“Long Survivor”Versus“Random” SCA	“Stroke”Versus“Long Survivor”	“Random” SCAVersus“Controls”
1p36.22	***CLCN6***	19	0.4686	**0.0080**	0.0527	1.0
1p21.1	***COL11A1***	69	**0.006066**	0.6627	0.1376	1.0
1q24.2	*F5*	12	0.5863	0.5849	0.0287	0.912
1q25.1	***SERPINC1***	21	0.0716	0.033	**0.017**	1.0
1q32.1	***ATP2B4***	13	**0.0082**	**0.0045**	0.0554	0.04
2p23.1	*CAPN13*	11	0.4759	0.0223	0.1827	0.812
2p13.1	***SLC4A5***	87	0.0462	**0.0061**	0.0505	0.023
2q34	***CPS1***	5	0.0737	0.7004	0.0767	0.021
2q37.3	***COL6A3***	9	**0.0104**	0.6201	0.0398	0.001
5p15.31	***MTRR***	3	0.2059	**0.0119**	0.02263	1.0
5q13.3	*HMGCR*	9	0.3361	0.4565	0.0453	0.062
5q31.1	***SLC22A5***	17	0.0478	0.6778	**0.0041**	0.011
7q21.11	***HGF***	45	0.0257	**0.0063**	0.0174	0.041
7q36.1	*NOS3*	39	0.342	0.0324	0.6525	0.76
8p11.21	***PLAT***	21	0.4267	**0.012**	0.4917	0.058
8q24.12	*SNTB1*	23	0.0228	0.354	0.2357	0.049
10p14	*GATA3*	12	0.2278	0.0356	0.3434	1.0
11q13.4	***NADSYN1***	130	**0.0172**	0.045	0.0247	0.017
14q11.2	***SLC7A8***	34	0.568	**0.0061**	0.05819	0.719
14q32.13	*SERPINA1*	78	0.0392	0.2231	0.0665	0.033
15q21.1	*SLC12A1*	97	0.0372	0.0848	0.5735	0.013
16p13.3	***CACNA1H***	134	0.0534	0.0229	**0.0139**	0.012
16p13.11	***ABCC1***	209	**0.0083**	**0.0155**	0.653	0.0246
17p12	***COX10***	49	0.3804	**0.0143**	0.3224	1.0
17q21.31	***SLC4A1***	156	0.5027	0.472	**0.007**	0.091
17q23.2	*MARCH10*	329	0.0375	0.0706	0.0287	0.028
19p13.3	***ABCA7***	278	0.194	**0.0151**	0.0884	0.078
19p13.2	***INSR***	178	**0.0106**	0.4731	0.0261	0.0191
20p11.21	*PYGB*	376	0.0257	0.043	0.0282	0.027

Table 3 shows all genes significant at a permutation *P* < .05 for at least one of the primary comparisons. Genes and *P*‐values significant at adjusted *P* < .017 are in bold. Numbers of individuals are: (a) “Stroke’ versus “Random” SCA (n = 79), (b) “Long Survivor” versus” Random” SCA (n = 82), (c) “Stroke” versus” Long Survivor” (n = 59). “Random” SCA versus “Controls” (n = 114) included as comparison

#### 3.1.2 Replication of mutational burden of genes in an independent sample

The characteristics of the replication cohort (including clinical events and hematological indices) are shown in Table S2. In the replication exome dataset, we identified 35 genes with mutations with functional impact as previously defined (Table S3). Twelve genes from the discovery analysis were replicated (Figure 2A; Table 2). Three genes (*HGF, SNTB1*, and *SERPINC1 [MIM: 107300]*) were found in the “stroke” group but not in the “long survivor” group (Figure 2A). Conversely, five genes (*CLCN6*, *OGDHL*, *COL6A3 [MIM: 120250]*, *INSR [MIM: 147670]*, and *NOS3 [MIM: 163729]*) were found in the “long survivor” group but not in the “stroke” group. Two genes (*NADSYN1* and *CACNA1H*) were common to all three groups (Figure 2A).

#### 3.1.3 Pathways and biological processes associated with genes with high mutational burdens

The PPI network formed from 17 genes containing high mutational burdens among the “long survivor” group was enriched for glutamate metabolism (*P *= .0035; Figure S4) and clustered with the fetal liver cell type (*P *= .0087) but had no association with any human disease/disorder. The PPI network of 19 genes in the “stroke” group was enriched for the arginine biosynthesis (*P *= .00078; Figure S5B), showed an association with hypertension (*P *= .00781), and clustered with monocytes (*P *= .029). The set of 39 genes with mutations identified from the “random” group (including the genes in common with the “long survivor” and “stroke” groups) was enriched for complement and coagulation cascades (*P *= 1.129 × 10^−6^; Figure S6B), associated with cholesterol level, diabetes mellitus, and thrombophilia (*P = *.00103, .0072, and .0095, respectively), and clustered with the adrenal cortex (*P *= .00018). The three sets of mutations from all 105 Cameroonian SCA patients (Figure 3A; Table S1) formed a network (Figure 3B) through gene hubs including *HGF*, *PLAT [MIM: 173370]*, *F5 [MIM: 612309]*, *F2 [MIM: 176930]*, *ESR2*, *INSR*, *RXRG [MIM: 180247]*, *PMS1 [MIM: 600258]*, and *MYOC [MIM: 601652]*. These gene hubs were associated with blood clotting cascade, vitamin B12 metabolism, and thrombophilia (*P *= .0000025, .000039, and .00029, respectively; Figure 3B,C). In the replication sample, we found enrichment of genes in glutamate metabolism, response to oxidative stress, complement/coagulation, and hemoglobin synthesis (Figure S7**)**. In the replication sample, novel findings that were not seen in the discovery samples were enrichment of genes in the following pathways: focal adhesion, angiogenesis, immune response and inflammation, hemoglobin production, longevity, nitric oxide, calcium signaling, and heme metabolism.

#### 3.1.4 Gene‐set allele frequency differentiation among groups of SCD patients and controls

We tested for gene‐specific differences in SNP frequencies among the three SCA groups (Figure 2B, Figures S8 A, B, and C). Comparing the “long survivor” and “stroke” groups, we found six genes exhibiting significant differentiation, including *VKORC1* ([MIM: 608547] *p *= 5.44 × 10^−7^), *FGA* ([MIM: 134820] *P *= 3.40 × 10^−6^), *FGFR3* ([MIM: 134934] *P *= 3.45 × 10^−6^), *PIGG* ([MIM: 616918] *P *= 8.64 × 10^−6^), *HFE2* ([MIM: 608374] *P *= 1.16 × 10^−5^), and *P1BA* (*P *= 1.83 × 10^−5^) (Figure S8C). These genes were clustered in complement and coagulation cascades (*P *= .00007; Figure 2A) and expressed in the liver (*P *= .0054). Comparing the “long survivor” group and the rest of the SCA patients (ie, “stroke” and “random”), three genes exhibited unusual differentiation (Figure S8B), including *CYP21A2*, *P2RY2 [MIM: 600041]*, and *PLAT* (*P *= 6.28 × 10^−6^, 1.22 × 10^−5^, and 1.97 × 10^−5^, respectively), enriched for blood coagulation (*P *= .0013) (Figures S9A, and S9C), associated with thrombophilia (*P *= .006818), and expressed in the liver (*P *= .0053). These genes are also implicated in fibrinolysis and the response to oxygen levels (GO:0042730 and GO:0070482, respectively; Figure S9C).

Comparing the “stroke” group and the rest of the SCA patients, we found frequency differences in *ITGA2B [MIM: 607759]*, *CALCA [MIM: 114130]*, *LTC4S [MIM: 246530]*, *EMILIN1 [MIM: 130660]*, *SERPINC1*, *NPHS1 [MIM: 602716]*, *ACD [MIM: 609377]*, and *USP37* (*P *= 3.46 × 10^−7^, 5.36 × 10^−7^, 3.96 × 10^−6^, 4.96 × 10^−6^, 6.22 × 10^−6^, 5.85 × 10^−6^, 1.86 × 10^−5^, and 1.48 × 10^−5^, respectively; Figure S8). These genes were enriched for blood coagulation (*P *= .00131), and glycosylphosphatidylinositol anchor biosynthesis (*P *= .0014), associated with rheumatoid arthritis (*P *= .046) and Fanconi anemia (*P *= .0467), and expressed in the liver (*P *= .0053). In the comparison of the controls against each patient group including “random,” “stroke,” and “long survivor” groups, we found *PLAT* and *SLC19A2* [MIM: 603941] to be consistently significant in gene‐specific differences in allele frequencies across all the comparisons (Table S4). A total of 29, 27, and 23 genes exhibited significantly unusual gene‐specific differences in allele frequency with nominal *P*‐values ranging from 2.5 × 10^−5^ to .05, in “random,” “stroke,” and “long survivor” patients’ groups against the Cameroon controls, respectively (Table S4). These genes are enriched with several genes identified with recurrent putative deleterious variants (Table 2 and Table S1) and those that clustered in hubs of an interaction network (Figure 2; Figures S4, S5, and S6). Specifically, *HBG2* [MIM: 142250] featured significant differentiation in allele frequencies with all SCD patients (*P *= 1.31 × 10^−5^, Table S4).

#### 3.1.5 Gene‐set rare‐variant association analyses

The main findings from the rare variant association tests are summarized in Table 3. We found 19 gene sets that were significantly associated at an adjusted *P* < .017 in the comparison of “stroke” versus “random,” “long survivor” versus “random,” and/or “long survivor” versus “stroke” (Table 3, Figure 3). Gene sets significantly associated with stroke included *ATP2B4, COLL11A1, COL6A3, NADSYN1, ABCC1 [MIM: 158343]*, and *INSR* while the gene sets significantly associated with long survival included *CLNC6, SLC24A5 [MIM: 609802], MTRR [MIM: 602568], HGF, PLAT, SLC7A8, ABCC1, COX10 [MIM: 602125]*, and *ABCA7 [MIM: 605414]*. One gene, *ATP2B4*, was associated with both “stroke” and “long survivor” groups. For the extreme contrast of “stroke” versus “long survivor”, only four gene sets – *SERPINC1, SLC22A5, CACNA1H*, and *SLC4A1* [MIM: 109270] – were significant. Notably, these four genes were not significant in the stroke association or long survival association tests, indicating that additional information was gained from this comparison.

The significant gene sets in the rare variant association analyses included most of the genes found to harbor recurrent deleterious variants in both the discovery and replication cohort (Table 2) and/or showing unusual allele frequency distributions (Table S4).

#### 3.1.6 Comparison with GWAS of sickle cell anemia and related traits

To contextualize our findings against the results of GWAS that have been conducted for sickle cell anemia and related traits, we queried the NHGRI‐EBI GWAS Catalog for all associations related to “sickle cell anemia” (EFO ID: Orphanet_232) and “haemoglobin F” (EFO ID: EFO_0004576). None of the genes significant in the present study (Tables S3 and S4) was significant in the relevant GWAS and vice versa. Therefore, our results represent findings unique to the exome space and not found by GWAS. We also note that there is no overlap between the genes in this study and genes significantly associated with HbF by GWAS. This observation implies that the effect of the genes we found to be significantly associated with long survival or stroke in this study are not mediated via HbF levels (one of the strongest and most consistently associated SCA modifiers).

#### 3.1.7 Functional support for identified pathways from transcriptomic studies

Gene expression provides functional evidence that a pathway is dysregulated in a disorder. To this end, we queried the significant pathways identified by sequence analysis in the present study against the most differentially dysregulated pathways in the two largest studies of global gene expression profiles in relation to SCD severity.^22^ Our findings show that most of our significant pathways also show significant transcriptomic differences in relation to SCD severity (Table 4), thus providing supportive evidence that these pathways are important in SCD pathophysiology.

**TABLE 4:** Significant pathways in this study that are also significant in global transcriptomic studies of SCD^21^, ^22^

	Transcriptomic evidence			
Pathway identified in the present study	Lowest *P*‐values in the present study	Associated phenotypes	*P*‐values in original studies	Source
Starch and sucrose metabolism	2.6 × 10^−9^	molecular risk profile	1.1871	^21^
One carbon pool by folate/folate pathway	5.8 × 10^−6^	molecular risk profile	3.3263 × 10^−8^	^21^
Complement and coagulation cascades	3.9 × 10^−8^	molecular risk profile	2.73693 × 10^−10^	^21^
Complement and coagulation cascades	3.9 × 10^−8^	acute crisis in children	.016	^22^
Oxidative Stress	1.8 × 10^−6^	top severity score in children	.00442	^22^
Oxidative Stress	1.8 × 10^−6^	acute crisis in children	5.6 × 10^−4^	^22^
Heme biosynthesis	1.7 × 10^−10^	top severity score in children	.00925	^22^
Heme biosynthesis	1.7 × 10^−10^	acute crisis in children	.005	^22^
Regulation of cellular response to stress	1 × 10^−09^	top severity score in children	.00008	^22^
Colorectal cancer/DNA repair system	.0002	molecular risk profile	1.51002 × 10^−12^	^21^

## 4 DISCUSSION

Our study addresses the issue of genetic modifiers of clinical variation in SCA in SSA using a whole‐exome sequencing approach. We utilized a design that included “long survivors” (representing patients surviving to the fifth decade despite the harsh environment and lack of state‐of‐the‐art medical care) and overt stroke patients (representing one of the most severe complications of SCA). By including a “random” comparison group, we provided a reference comparison group of the “average” SCA patient. This phenotype grouping that explicitly recognizes that some complications take time to emerge (eg, stroke‐ or age‐related complications/mortality) is a methodologic approach that, perhaps, could be considered innovative and a strength of this manuscript. Given that there were no significant findings among the groups for fetal hemoglobin levels, classical sickle haplotypes, and α‐thalassemia, our study implicitly controlled for these known factors for clinical heterogeneity in SCD. However, the lack of significance of HbF levels, a well‐established and strongest known modifier of SCD childhood complications and that is influenced by genomic variations and therapeutic interventions, probably reflects the fact that the sample studied have all survived past the “under‐5‐year‐old” mortality hazards (malaria, bacterial sepsis, diarrheal disease, or splenic sequestration, etc.).

The main findings point to different gene sets that are enriched for deleterious and loss‐of‐function mutations in phenotypically defined groups of patients and with evidence of genetic association with different phenotypes, providing support for the complexity of the genetic architecture of SCD phenotypic variability. Notably, pathways represented by these genes point to relevant pathophysiological mechanisms, including some that are already therapeutic targets. Our findings of the involvement of glutamine (*NADSYN1*) and arginine (*OGDHL* and *NOS3*) are novel and noteworthy. Decreased erythrocyte glutamine levels contribute to alterations in the erythrocyte redox environment and hemolysis and play a role in the pathogenesis of pulmonary hypertension in SCD.^23^ l‐Glutamine was recently approved by the US FDA as a medication for SCD.^24^ A non‐synonymous variant in the arginine‐fifty homeobox gene (*ARGFX*) was previously associated with stroke in SCD.^13^ Low‐dose supplementation with l‐arginine improved liver function, oxidative stress, nitric oxide metabolite levels, cardiovascular dysfunction, and sickle cell‐related pain.^25^ *CACNA1H* is associated with hypertension and is therapeutically targetable by calcium channel blockers.^26^ Thus, our findings using sequence analysis of African SCA patients has support from prior studies. Moreover, the findings of vascular and NO signaling variants are consistent with the clinical observations that long term survival among African Americans patients are dependent on vasculopathic complications.^27^

A major finding of this study is the observation that “long survivor” group was characterized by mutational burdens in *CLCN6* and *OGHDL*. Rare, deleterious mutations in *CLCN6* (a voltage‐dependent chloride channel) have been associated with lower blood pressure.^28^ Given that increased blood pressure is a major risk factor for stroke in SCD,^29^ this suggests that SCD patients with *CLCN6* mutations live longer due to a reduced risk of stroke. *OGHDL* is important in arginine metabolism, which is a key factor in the hemolysis‐endothelial dysfunction observed in SCD and has become a target for therapeutic interventions as noted above. Interestingly, in a recent trial in patients with SCD, the median number of pain crises over 48 weeks was lower among those who received oral therapy with l‐glutamine.^30^ Variants in genes involved in complement and coagulation cascade and fibrinolysis appear to be important for all SCD patients and particularly for susceptibility to stroke. It is well known that SCD involves a hypercoagulable state.^3^, ^13^ *SERPINC1* encodes antithrombin, implicating loss of SERPINC1 activity with increased blood coagulation. Transcriptomic expression of complement and coagulation components in circulation was increased in a cluster of African American SCD patients with higher severity and mortality rate.^21^, ^22^ Moreover, there is evidence of increased complement activation in older patients with SCD,^31^ and a complement gene *C5* mutation was associated with stroke in SCD patients.^13^ In summary, the evidence from this and other studies suggest that long survival is characterized by mutations that confer protection for adverse phenotypes (notably stroke) given that these genes influence intermediate phenotypes for stroke (including blood pressure and endothelial function) while the overt stroke phenotype in SCA is associated with mutations in genes that are involved in the complement and coagulation cascade. Identification of genes such as *LTC4S*, that displayed specific signal of unusual difference in SNPs frequencies among patients with stoke (Figure S8A), will deserve future investigations with appropriate experimental design to explore other genes involved in leukotriene synthesis pathways, that have long been associated with SCD pain, airway hyperresponsiveness, and hospitalization rates.^32^, ^33^ Specifically, future studies should investigate leukotriene levels as potential marker for stroke, in as much as leukotriene antagonists are being tested in Phase 2 trial for SCD‐related comorbidities in SCD.^34^

Long survival in SCA is a composite phenotype that includes factors that decrease mortality‐causing events (eg, strokes) and/or are associated with improved indices of health (eg, favorable blood pressure and lipid profiles), which in turn have their own risk factors. While the present study was not designed to measure most of these intermediate phenotypes, we note that the genes with high mutational burden found in the present study were annotated to functional pathways related to some of the intermediate phenotypes and also show association with severity phenotypes in independent transcriptomic studies (as shown in Table 4). Further studies are needed to investigate the mechanisms by which these mutational changes influence the phenotype. We also recommend that future longitudinal studies with larger sample sizes, from diverse population groups and settings in Africa, Europe, and America, should include HbF levels, blood pressure, markers of oxidative stress, arginine/ glutamine levels, blood coagulation markers, and markers of heme pathways/hemolysis as important clinical variables for genotype‐phenotype association, in relation to long‐term survival.

Involvement of genes in the pathways of vitamin B12 and folate metabolism is expected as these pathways are important for regulation of erythropoiesis. Subjects with SCD are at higher risk of cobalamin deficiency, justifying supplementation in clinical practice.^35^ Identification of genes involved in mitotic check‐point and DNA repair, starch, and sucrose metabolism, and solute carriers require further study to explore their roles in modifying the SCD phenotype. Similarly, future studies on heme pathways can explore if hemolysis metabolism or susceptibility are responsible for findings in this signaling hub in the replication samples.

To our knowledge, this is the first investigation of clinical variation in SCD in Africa using a whole‐exome sequencing approach. Strengths of the study include well‐defined clinical groups, inclusion of an independent replication sample, study sites where treatment is unlikely to confound outcomes, use of several different but complementary analytical approaches and linking the identified genes and pathways to published transcriptomic and therapeutic data. Nonetheless, the study has some limitations. The stroke group consisted of patients with overt stroke and would therefore not have captured patients with silent cerebrovascular events. In addition, we focused on overt stroke, without brain imaging than could have further sub‐stratified this phenotype as ischemic or hemorrhagic, and identified subclinical infarcts, that are also found in SCD in children in Africa.^36^, ^37^ These sub‐classifications could have allowed differential exploration of genetic protective and pathophysiologic risk factors, for example, variants in genes in hemolysis pathways for ischemic stroke, and variants in genes of vasculopathy, hypertension, and connective tissues pathways for hemorrhagic stroke. However, it should be noted that our focus is on a severe event (overt stroke), not on silent subclinical events, which, by definition, do not represent severe clinical events. The ideal study design for outcomes in SCA is a longitudinal study. In its absence, we have used a “random group” in the present study to enable us to distinguish between genes with similar mutation burden in all SCD patients irrespective of the clinical severity versus those genes that exhibit such characteristics in specific extreme groups, such as “long survivor” and “stroke” patients. Future studies with a longitudinal design will indeed provide a better comparison than what is possible with a cross‐sectional study. Also, the sample sizes are relatively modest and larger sample sizes would probably yield more findings, as illustrated by the finding of additional genes and pathways found in the replication group but not in the discovery group. Nonetheless, the total number of exomes sequenced in the study represents one of the largest (and only available from Africa) such datasets for SCA severity to date.

In summary, we reported a WES study on clinical phenotypes of SCA in Africa. We generated a catalogue of candidate modifier genes that clustered in pathophysiological pathways important in SCA and with implications for therapeutic intervention. This study fills an important gap in knowledge by using a WES approach focusing on deleterious coding variants important in two specific clinical categories of SCA patients (long survival and overt stroke), in contrast to most other studies that used a GWAS approach and often used fetal hemoglobin levels as a proxy of severity. This study thus makes significant contributions to present knowledge of the natural history and clinical heterogeneity of SCA in SSA, with the potential for informing the design of new therapeutics.

## 5 WEB RESOURCES

Online Mendelian Inheritance in Man: http://www.omim.org

SKAT: SNP‐Set (Sequence) Kernel Association Test: https://cran.r-project.org/web/packages/SKAT/index.html

6

## CONFLICT OF INTEREST

The authors declare no competing interests. The authors alone are responsible for the content and writing of this article.

## Supporting information

Figure S1. Workflow of the data analysis.
Figure S2. SCA exome map quality. A, The Venn diagram shows the overlap of variants between three variant caller methods used: GATK, samtools, and freebayes. B, Overall depth distribution of SCA exome map. C, Overall percentage of variant functions from 8,458,386 variants. D, Number of heterozygotes by allele frequency. E, Substitution types. F, Ts/Tv by allele frequency.
Figure S3. Comparison of SCA exome map with 1000 Genomes Project data. A, Principal component analysis of the three Cameroonian SCA sub‐groups (“random”, “stroke”, and “long survivor”) and continental African samples from the 1000 Genomes Project phase 3 release. Both components show a closer relationship between the SCA and Africans than non‐Africans. A, Overall percentage of exonic variant functions of 8,458,386 variants. C, Principal component analysis of the three SCA groups, showing a slight departure of “long survivor” to the other SCA patient groups.
Figure S4. Biological sub‐network of the identified candidate mutations in “long survivor” SCA patients. A, Sub‐network of the identified candidate mutations in the “long survivor” SCA group. B, Diagram of the top significant pathways associated with the identified candidate mutations.
Figure S5. Biological sub‐network of the identified candidate gene mutations in the “stroke” SCA group. A, Sub‐network of the identified candidate mutations in the “stroke” SCA group. B, Diagram of the top significant pathways associated with the identified candidate mutations.
Figure S6. Biological sub‐network of the identified candidate mutations in “random” SCA group. A, Sub‐network of the identified candidate mutations in the “random” SCA group. B, Diagram of the top significant pathways associated with the identified candidate mutations.
Figure S7. Biological sub‐network of the identified candidate gene mutations in a replication cohort of 29 SCA patients from DRC. A, Sub‐network of the identified candidate mutations. B, Diagram of the top significant pathways associated with the identified candidate mutations.
Figure S8. Circular Manhattan plot of gene‐specific signals of unusual difference in SNPs frequency. A, Unusual gene‐specific allele frequency differences between the “stroke” group and the rest of the SCA patients. B, Unusual gene‐specific allele frequency differences between the “long survivor” group and the rest of the SCA patients. C, Unusual gene‐specific allele frequency differences between the “stroke” and “long survivor” groups.
Figure S9. Significant pathways and biological processes associated with genes that differentiate “long survivor” and other SCA patients. A, Significant pathways associated with genes exhibiting unusual gene‐specific allele frequency differences between the “long survivor” group and other SCA patients. B, Diagram of the top significant pathway in panel A. C, Significant biological processes associated with genes exhibiting unusual gene‐specific allele frequency differences between the “long survivor” group and other SCA patients.
**Table S1**. Genes with significant mutations in SCA patients from Cameroon (See details in Table S5 of mutations within reported genes).
**Table S2**. Characteristics of the replication cohort (SCA patients from Democratic Republic of Congo)
**Table S3**. Genes with significant mutations in SCA patients from Democratic Republic of Congo (DRC).
**Table S4**. Gene‐specific signal of unusual difference in allele frequencies between Cameroon control versus all SCA, “random”, “stroke”, and “long survivor” SCA patients.
**Table S5**. Details of mutations identified within genes (Table S1) in SCA patients from Cameroon.

## Data Availability

The datasets used and/or analysed during the current study are available from the corresponding author on reasonable request.
