# Supplementary material for: Genetic modifiers of long‐term survival in sickle cell anemia
Source: Clin Transl Med. 2020 Aug 12;10(4):e152. doi: 10.1002/ctm2.152 (PMC7423184; doi:10.1002/ctm2.152)
Supplement: Supplementary file 1 — Figure S1. Workflow of the data analysis. Figure S2. SCA exome map quality. A, The Venn diagram shows the overlap of variants between three variant caller methods used: GATK, samtools, and freebayes. B, Overall depth distribution of SCA exome map. C, Overall percentage of variant functions from 8,458,386 variants. D, Number of heterozygotes by allele frequency. E, Substitution types. F, Ts/Tv by allele frequency. Figure S3. Comparison of SCA exome map with 1000 Genomes Project data. A, Principal component analysis of the three Cameroonian SCA sub‐groups (“random”, “stroke”, and “long survivor”) and continental African samples from the 1000 Genomes Project phase 3 release. Both components show a closer relationship between the SCA and Africans than non‐Africans. A, Overall percentage of exonic variant functions of 8,458,386 variants. C, Principal component analysis of the three SCA groups, showing a slight departure of “long survivor” to the other SCA patient groups. Figure S4. Biological sub‐network of the identified candidate mutations in “long survivor” SCA patients. A, Sub‐network of the identified candidate mutations in the “long survivor” SCA group. B, Diagram of the top significant pathways associated with the identified candidate mutations. Figure S5. Biological sub‐network of the identified candidate gene mutations in the “stroke” SCA group. A, Sub‐network of the identified candidate mutations in the “stroke” SCA group. B, Diagram of the top significant pathways associated with the identified candidate mutations. Figure S6. Biological sub‐network of the identified candidate mutations in “random” SCA group. A, Sub‐network of the identified candidate mutations in the “random” SCA group. B, Diagram of the top significant pathways associated with the identified candidate mutations. Figure S7. Biological sub‐network of the identified candidate gene mutations in a replication cohort of 29 SCA patients from DRC. A, Sub‐network of the identified candidate mutations. [file CTM2-10-e152-s001.pdf]

Supplemental reports

Contents

Supplemental figures and legends……………………………………………………………2
Figure S1………………………………………………………………………………………..3
Figure S2………………………………………………………………………………………..4
Figure S3………………………………………………………………………………………..5
Figure S4………………………………………………………………………………………..6
Figure S5………………………………………………………………………………………..7
Figure S6………………………………………………………………………………………..8
Figure S7………………………………………………………………………………………..9
Figure S8………………………………………………………………………………………10
Figure S9………………………………………………………………………………………11

Supplemental tables………………………………………………………………………….12
Table S1………………………………………………………………………………………..13
Table S2………………………………………………………………………………………..16
Table S3………………………………………………………………………………………..17
Table S4………………………………………………………………………………………..19
Table S5………………………………………………………………………………………..22

Supplemental methods……………………………………………………………………….33
Introduction……………………………………………………………………………………33
Library Construction and Sequence…………………………………………………………...34
Reads Mapping and Alignment………………………………………………………………..35
Variant Calling…………………………………………………………………………………36
Variant Calling Quality Control and Final Call-set……………………………………………36
Variant Annotation and Mutation Prioritization………………………………………………37
Network and Enrichment Analysis…………………………………………………………….38
Further Characterization of Enriched Sub-networks…………………………………………..39
Aggregating Summary Statistics at the Gene and Sub-network Level……………………….40
Phased and Haplotypes Inference……………………………………………………………...41
Fine-scale Population Structure………………………………………………………………..42

Supplemental references……………………………………………………………………..46

Supplemental figures and legends

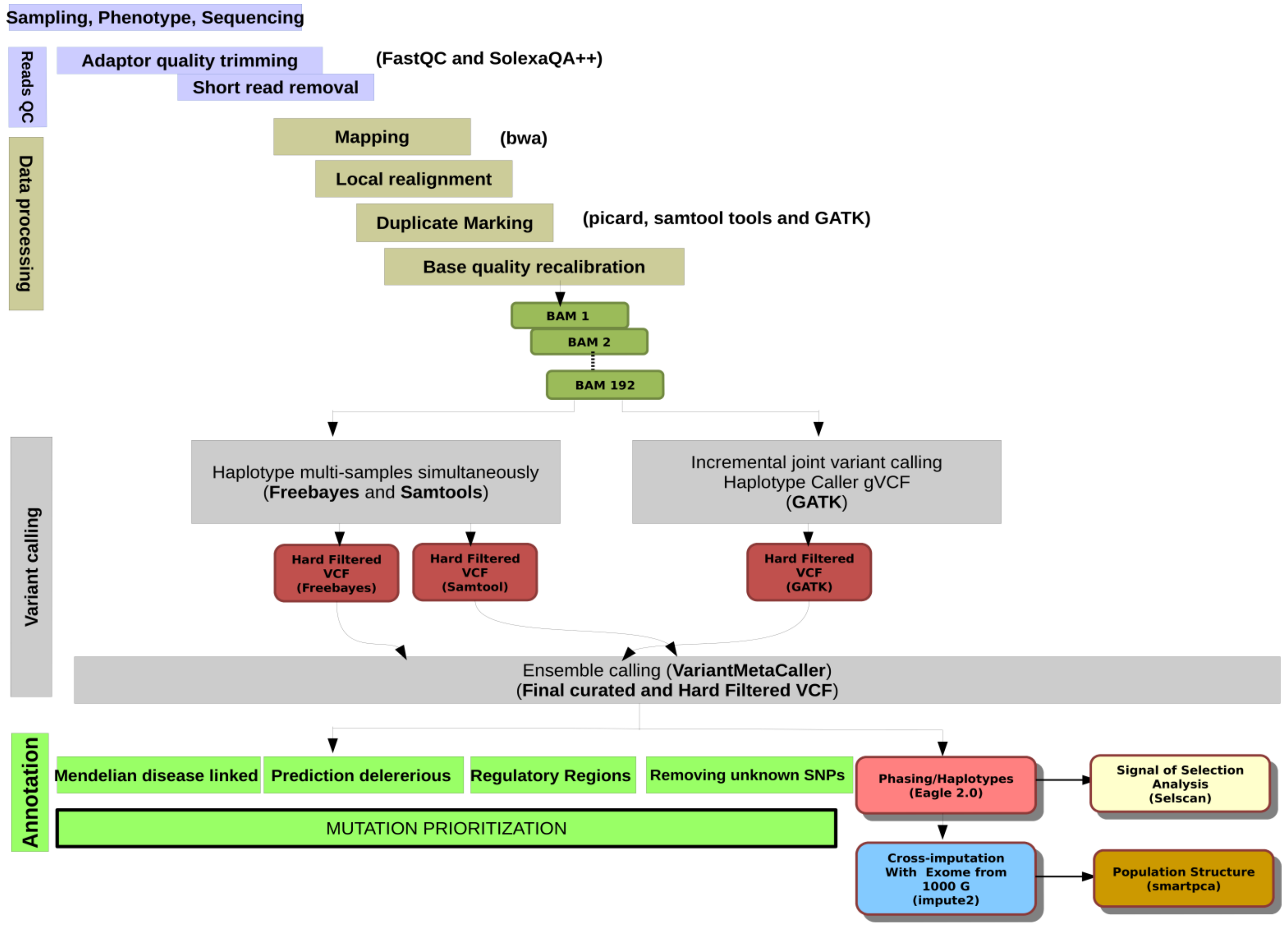

Figure S1. Workflow of the data analysis.

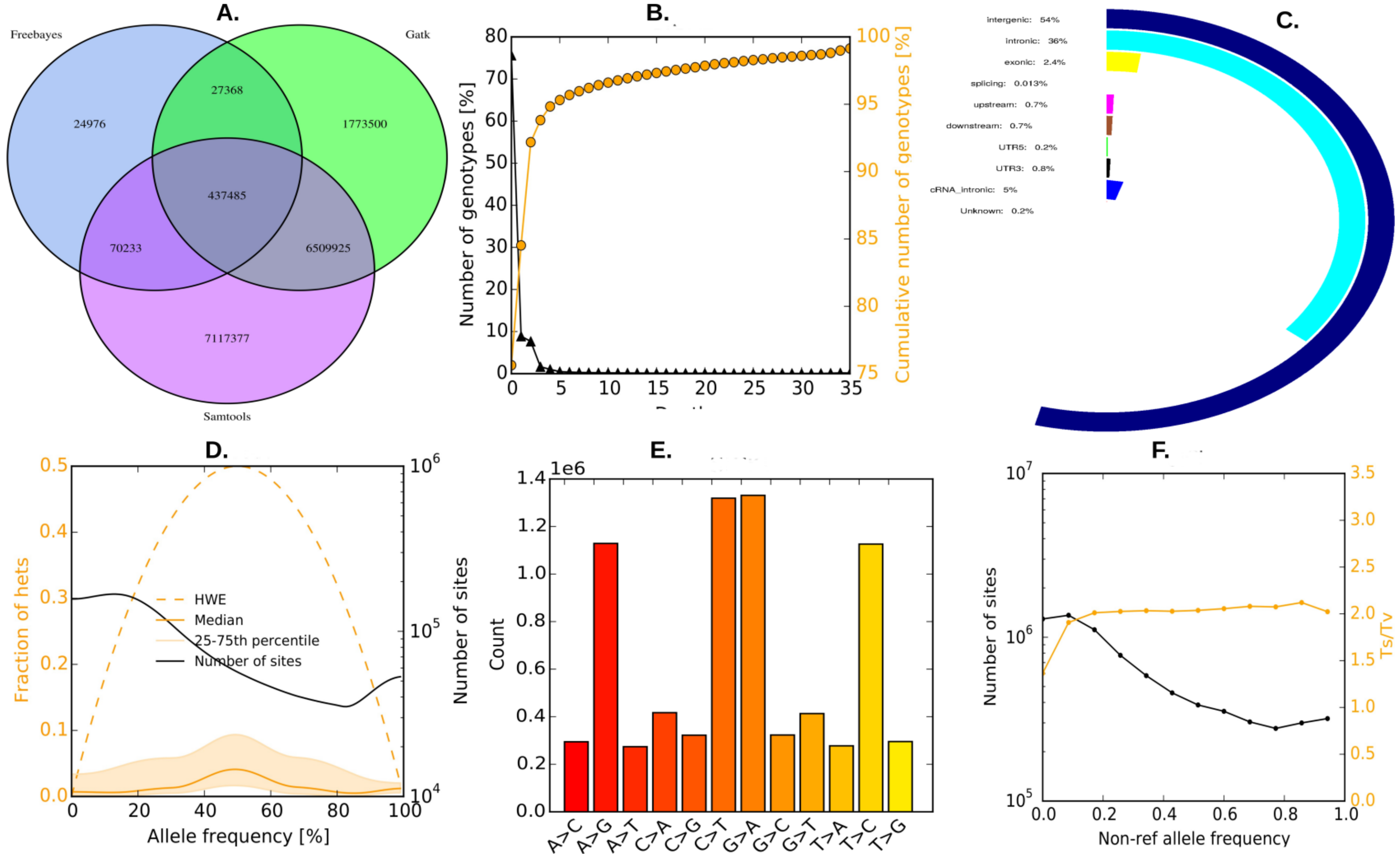

Figure S2, SCD exome map quality. A, the Venn diagram shows the overlap of variants between three variant caller methods used, gatk, samtool and freebayes. B, Overall depth distribution of SCD exome map. C, overall percentage of variant functions from 8,458,386 variants. D, Number of heterozygous by allele frequency. E, Substitution types. F, Ts/Tv by allele frequency.

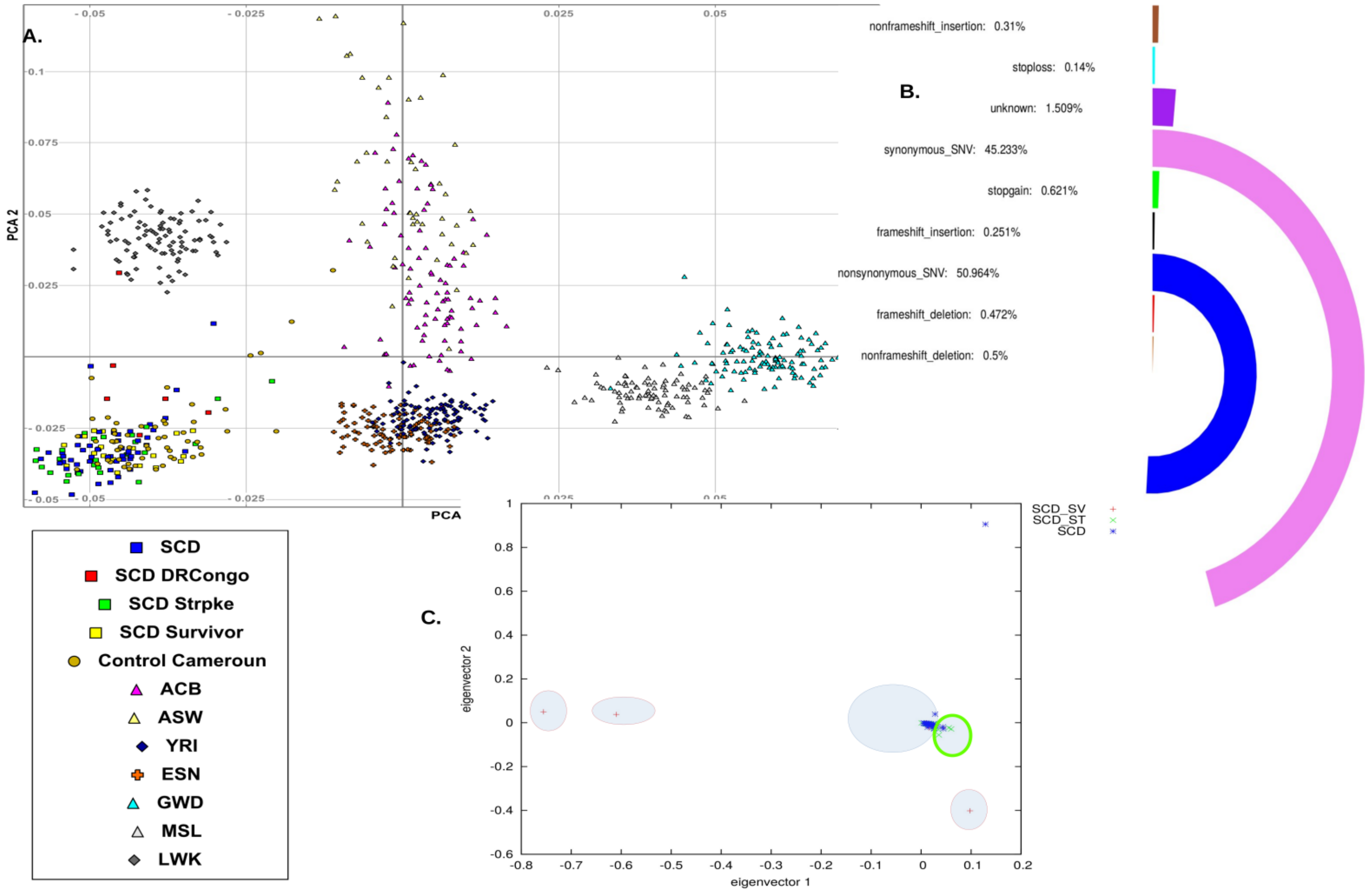

Figure S3. Comparison of SCD exome map with recently 1000 Genomes release. A, principal component analysis of the three Cameroonian SCD sub-groups ("Random", "Stroke" and "Survival"), and continental samples from 1000 Genomes phase 3 release. Both components show a close relationship between the SCD and Africans than non-Africans. B, overall percentage of exonic variant functions of 8,458,386 variants. C, principal component analysis of the three SCD groups ("random", "Stroke" and "Survival"), showing a slight departure of survival to the rest of other SCD patients. D, the Venn diagram shows the overlap of SCD exome map and 1000 Genomes release phase3.

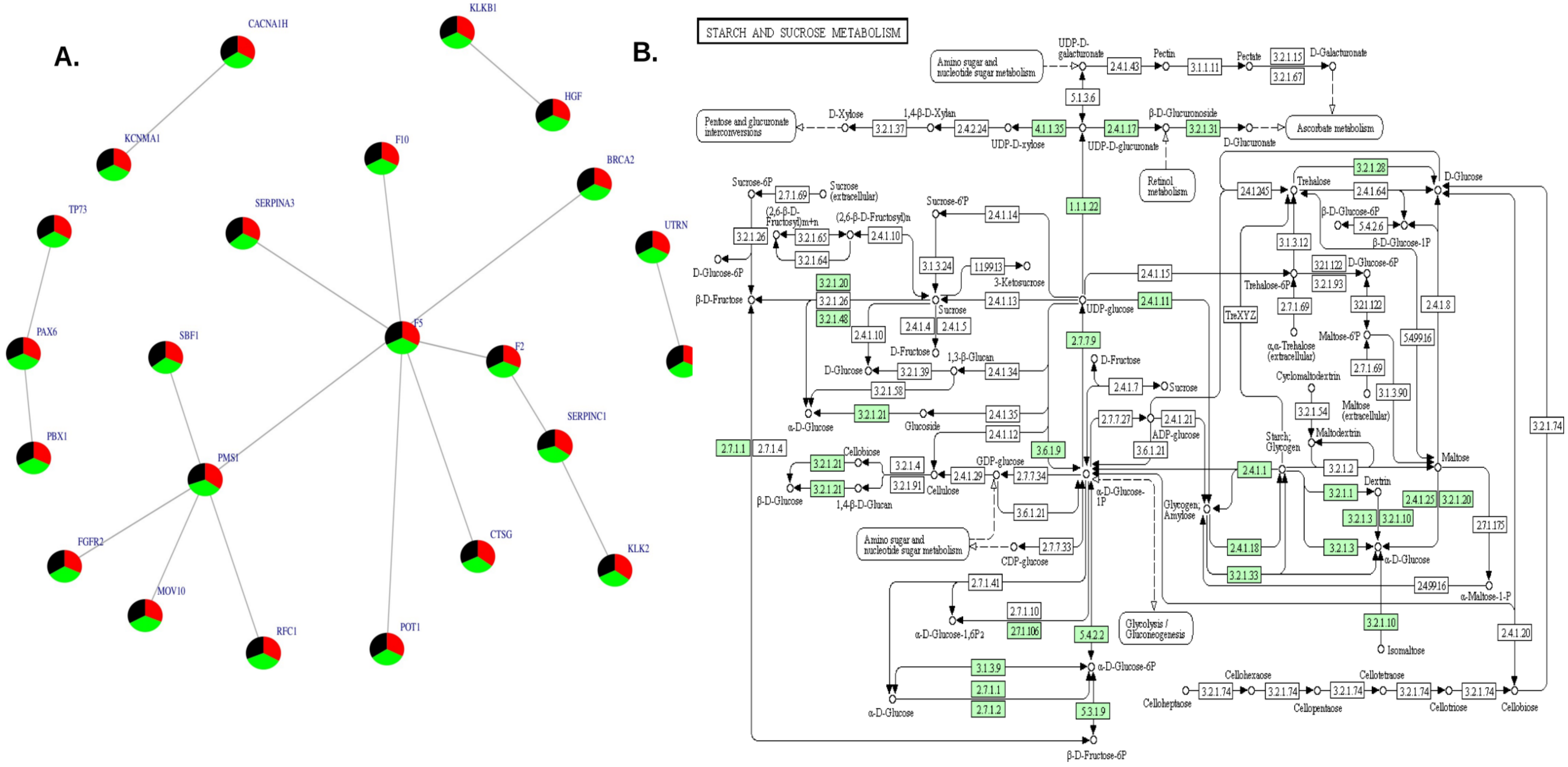

Figure S4. Biological sub-network of the identified candidate mutations in 'long survivor" SCD patients. A, sub-networks of the identified candidate mutation in the survival. B, diagram of the top significant pathways associated with the identified candidate mutations.

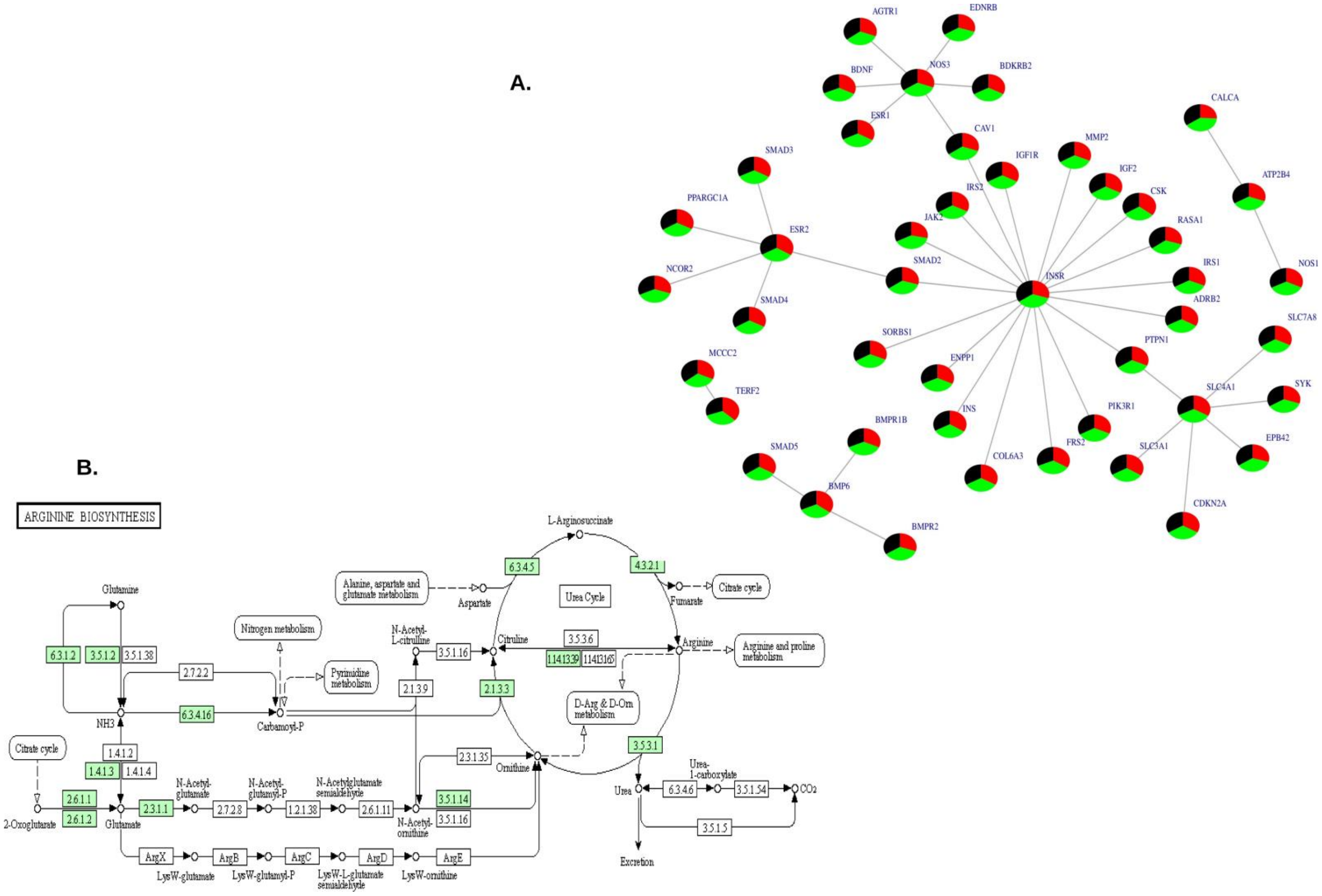

Figure S5. Biological sub-network of the identified candidate gene mutations in SCD patients with stroke. A, sub-networks of the identified candidate mutation in SCD with stroke. B, diagram of the top significant pathways associated with the identified candidate mutations.

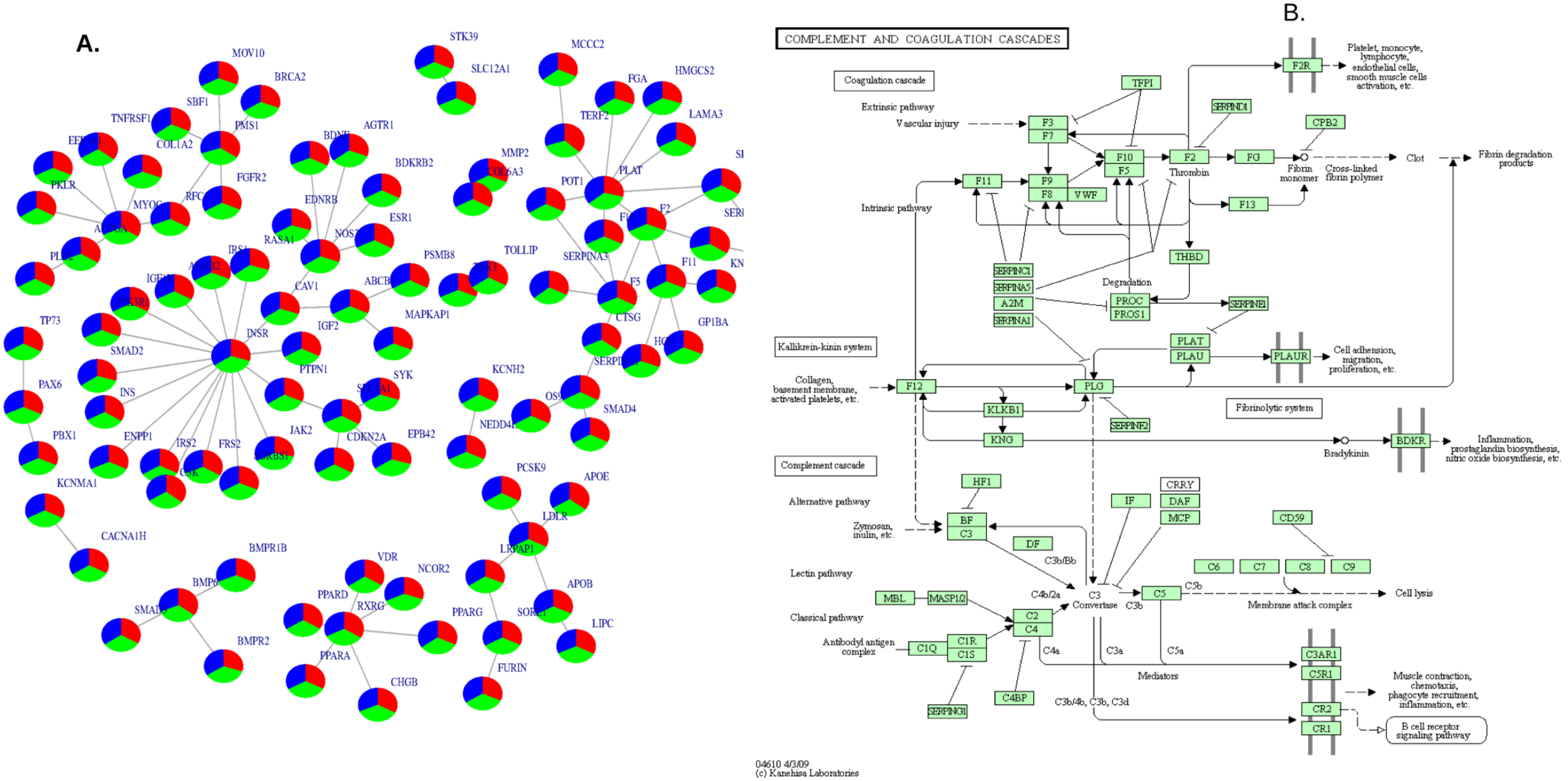

Figure S6. Biological sub-network of the identified candidate mutations in "random" SCD patients' group. A, sub-networks of the identified candidate mutation in the normal SCD. B, diagram of the top significant pathways associated with the identified candidate mutations.

A.

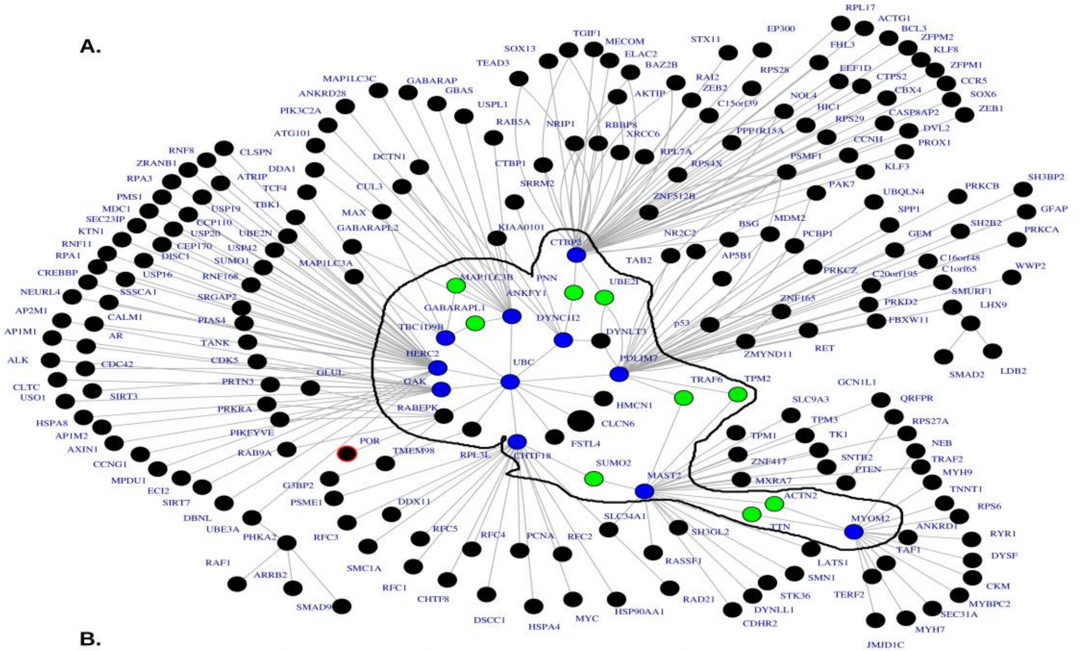

B.

Table: Pathways Associated to the network formed with genes interacting with identified mutation genes in SCD DRC (see Extended Table 2).

**Biological Pathways**	**Enrichment Pvalues**	**Adjusted Pvalues**	**Searched databases**
Focal adhesion	2.2e-21	1.1e-19	BioCarta, KEGG, NCI-Nature,Panther
Angiogenesis	1.7e-20	9e-15	BioCarta,KEGG,Panther
Actions of Nitric Oxide in the Heart	9.14e-09	3.5e-07	BioCarta, KEGG, NCI-Nature, Panther
Regulation of actin cytoskeleton	1e-109	1e-07	KEGG, Panther
Longevity regulating pathway	5.9e-09	2.8e-08	BioCarta, KEGG, NCI-Nature, Panther
Complement and coagulation cascades	3.9e-08	1.5e-07	KEGG, NCI-Nature, Panther
Oxytocin signaling pathway	1.8e-06	5.1e-06	KEGG, BioCarta
Calcium signaling pathway	5.8e-06	1.5e-05	BioCarta

Figure S7. Biological sub-network of the identified candidate gene mutations in SCD patients a replication cohort of 29 SCD patients from DRC. A, sub-networks of the identified candidate mutation. B, diagram of the top significant pathways associated with the identified candidate mutations.

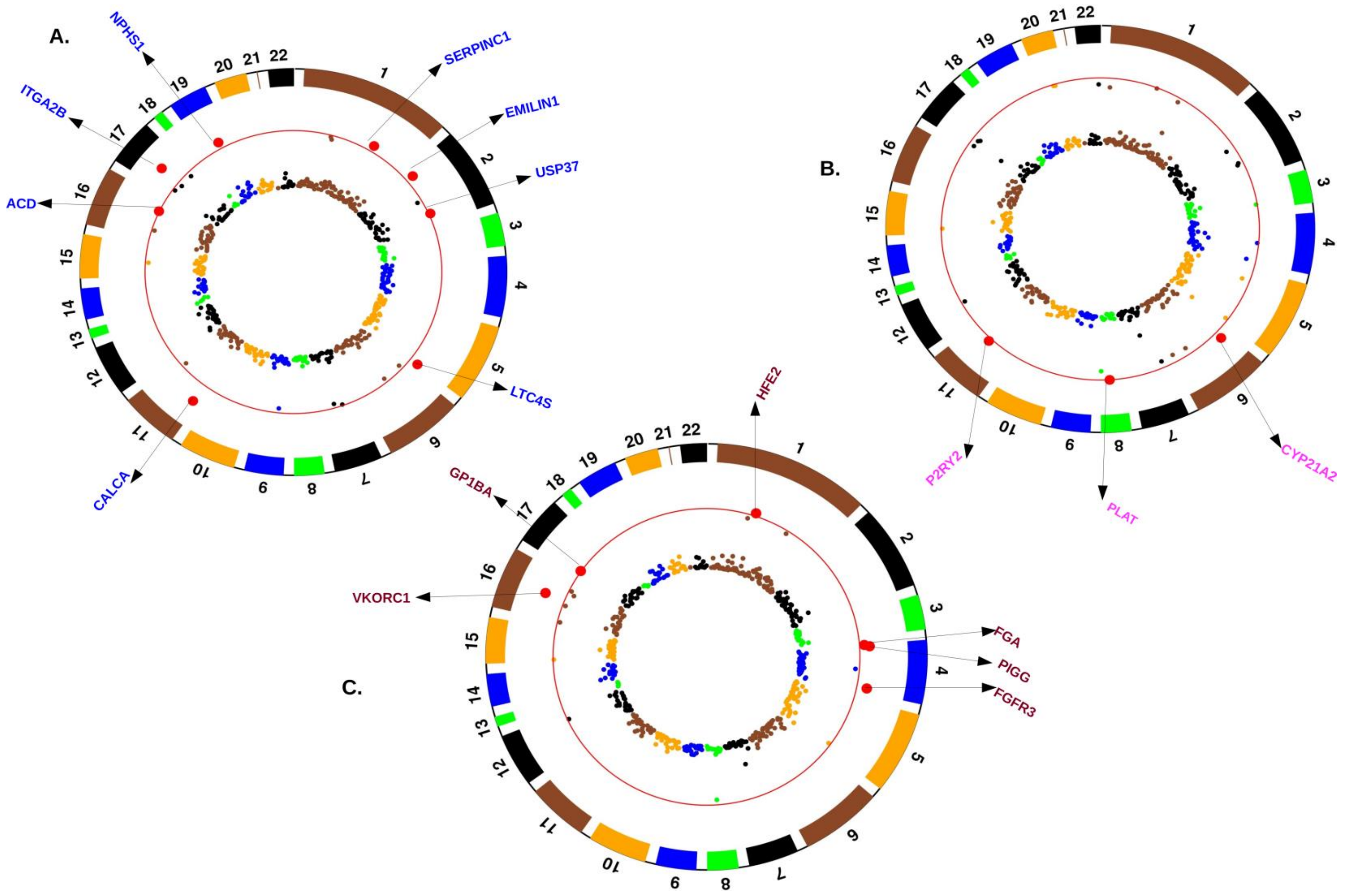

Figure S8. Circular Manhattan plot of gene-specific signal of unusual difference in SNPs frequencies. A, unusual gene-specific frequency difference between SCA with stroke, and the rest of SCA patients. B, gene-specific signal of unusual genetic difference between "long survivor" patients and the rest of SCA patients. C, unusual differentiation between SCA with stroke and "long survivor" SCA patients. **Table S3** displays the results of Cameroun control versus each the above SCA group.

A.

Significant human pathways associated to genes of large departure of unusual gene-specific difference between survival, and other SCD patients.

Term	P-value	Adjusted P-value	Z	Combined Score
Complement and Coagulation Cascades	0.00007	0.006	-1.91	9.61
Blood Clotting Cascade	0.0006	0.02	-1.91	7.30
TP53 Network	0.002	0.03	-1.77	5.72

C.

Significant human biological process associated to genes shown unusual gene-specific difference between survival, and other SCD patients.

Term	P-value	Adjusted P-value	Z-score	Combined Score
fibrinolysis (GO:0042730)	0.000006	0.006	-2.82	14.41
response to oxygen levels (GO:0070482)	0.00009	0.009	-2.49	11.73
response to decreased oxygen levels (GO:0036293)	0.00007	0.009	-2.48	11.67
response to hypoxia (GO:0001666)	0.00007	0.009	-2.47	11.66
mitotic G1/S transition checkpoint (GO:0044819)	0.0002	0.01	-2.68	11.33
G1 DNA damage checkpoint (GO:0044783)	0.0002	0.01	-2.65	11.22

B.

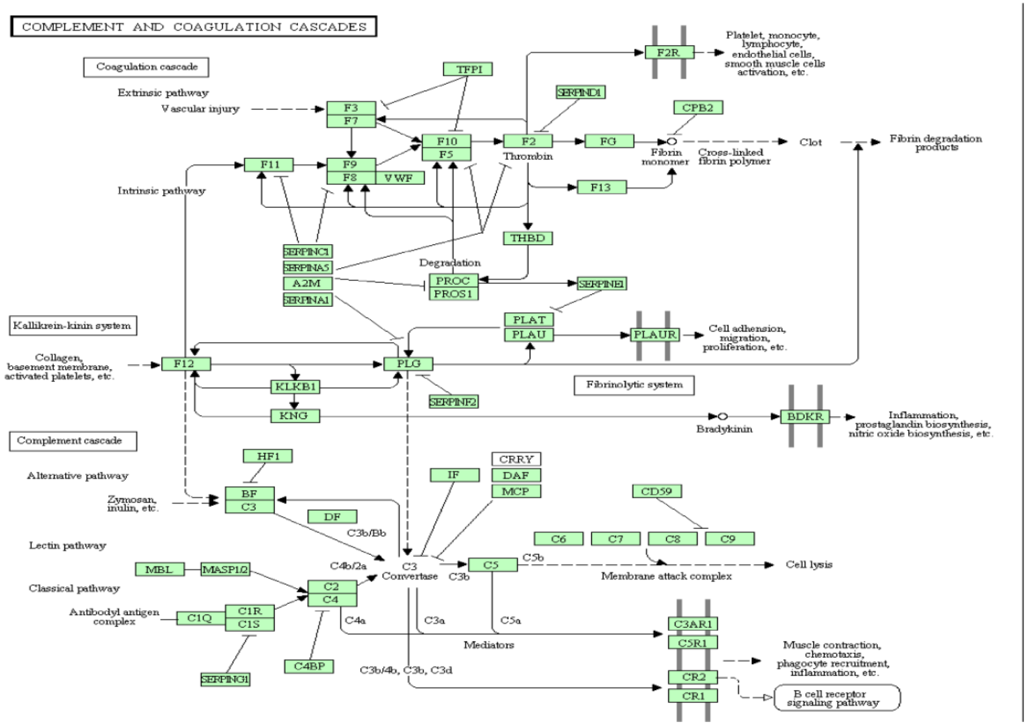

Figure S9. Significant pathways and Biological processes associated to gene-specific difference in SNPs frequency between "long survivor**" and other SCD patients**. A. Significant pathways associated to genes exhibited a large unusual gene-specific difference between "long survivor" group and other SCD patients. B. diagram of the top significant pathway in panel A. C. Significant biological processes associated to genes with unusual departure of gene-specific exome wide difference between "long survivor" and other SCD patients.

Supplemental Tables

Table S1. Genes with mutations in SCD patients from Cameroon. The Z-score is obtained from aggregating the SiPhy (29-way) score based on identified mutants SNPs within gene (See details in Table S4 of mutation in polymorphisms within reported genes). The reported Homozygous; heterozygous, compound heterozygous**,** cDNA change, Protein change, and all ExAc frequencies are from the polymorphism with top SiPhy (29-way) score.

CHR	Max # SV	Max # Patients	Region	Genotypes	Gene	Gene name	cDNA Change	Protein Change	ExAC AFR	ExAC EUR	"Random" SCD Z-score	SCD with stroke Z-score	"Long Survivor" SCD Z-score
2	11	69/105	2q34	Hom	*CPS1*	Carbamoyl-phosphate synthetase 1, mitochondrial	c.C1086G	p.V362V	0	0.01	20·86	20·86	20·86
2	7	82/105	2p13.1	Hom	*SLC4A5*	Solute carrier family 4, sodium bicarbonate cotransporter, member 5	c.C2790T	p.T930T	0·0003	0	15·65	15·65	15·65
11	12	83/105	11q13.4	Hom	*NADSYN1*	NAD synthetase 1	c.G256A	p.V86M	0	0	15·15	15·5	15·15
16	3	79/105	16p13.3	Hom	*CACNA1H*	Calcium channel, voltage-dependent, T type, alpha 1H subunit	c.G93A	p.E31E	0	0	15·05	15·18	15·05
20	2	76/105	20p11.21	Het	*PYGB*	Phosphorylase, glycogen; brain	c.G907T	p.A303S	0	0	18·71	18·71	18·71
1	3	47/82	1q25.1	Het	*SERPINC1*	Serpin peptidase inhibitor, clade C , member 1	c.A1011A	p.Q337Q	0	0	19·75	19·75	-
1	14	68/82	1q24.2	Het	*F5*	Coagulation factor V	c.2247_2249del	p.749_750del	-	-	17·62	17·62	-
2	8	41/82	2q32.2	Het	*PMS1*	PMS1 postmeiotic segregation increased 1	c.T141C	p.Y47Y	0	0	19·13	18·06	-
10	6	58/82	10p14	Het	*GATA3*	GATA binding protein 3	c.T606C	p.R202R	-	-	18·28	18·28	-
11	2	49/82	11p13	Het	*PAX6*	Paired box 6	c.C1139T	p.S380L	-	-	20·86	20·86	-
19	5	67/82	19q13.11	Het	*GPI*	Glucose phosphate isomerase	c.G7A	p.A3T	0	0	15·39	15·39	-
19	3	53/79	19p13.2	Het	*INSR*	Insulin receptor	c.C3255T	p.H1085H	0	0	15·83	-	15·83
7	7	66/79	7q36.1	Het	*NOS3*	Nitric oxide synthase 3	c.341_348del	p.S114fs	0	0	15·73	-	15·73
5	3	40/79	5q13.2	Het	*MCCC2*	Methylcrotonoyl-Coenzyme A carboxylase 2 (beta)	c.T969C	p.A323A	-	-	18·04	-	18·04

17	14	51/79	17q21.31	Hom	*SLC4A1*	Solute carrier family 4, anion exchanger, member 1	c.T2688C	p.D896D	-	-	19·270	-	18·79
5	2	38/79	5p15.31	Het	*MTRR*	5-methyltetrahydrofolate-homocysteine methyltransferase reductase	c.A147G	p.I49M	-	-	19·16	-	19·16
2	9	54/79	2q37.3	Hom	*COL6A3*	Collagen, type VI, alpha 3	c.G3446A	p.R1149Q	0	0	19·472	-	16·95
6	13	62/79	6p24.3	Hom	*BMP6*	Bone morphogenetic protein 6	c.566delC	p.A189fs	-	-	19·70	-	19·75
6	1	37/79	6q23.2	Het	*VNN1*	Vanin 1	c.T1540C	p.X514Q	0	0	20·64	-	20·64
5	8	15/26	5q31.1	cHet	*SLC22A5*	Solute carrier family 22 , member 5	c.280delG	p.A94fs	-	-	-	16·82	-
7	1	17/26	7q21.11	Het	*HGF*	Hepatocyte growth factor	c.T696C	p.H232H	0	0	-	19·91	-
8	3	13/26	8q24.12	Het	*SNTB1*	Syntrophin, beta 1	c.C1338T	p.C446C	0	0	-	19·27	-
15	1	11/26	15q15.1	cHet	*IVD*	Isovaleryl Coenzyme A dehydrogenase	c.C20T	p.A7V	-	-	-	18·85	-
16	7	9/26	16p13.11	cHet	*ABCC1*	ATP-binding cassette, sub-family C , member 1	c.C33T	p.G11G	0	0	-	19·56	-
1	14	19/26	1q32.1	Hom	*ATP2B4*	ATPase, Ca++ transporting, plasma membrane 4	c.A111A	p.S37S	0·0004	0	-	-	20·19
1	11	20/23	1p36.22	Hom	*CLCN6*	Chloride channel 6	c.G234A	p.A78A	0	0	-	-	19·058
10	5	21/23	10q11.23	Hom	*OGDHL*	Oxoglutarate dehydrogenase-like	c.G2931A	p.A977A	0	0	-	-	19·95
14	2	19/23	14q23.2	Het	*ESR2*	Estrogen receptor 2	c c.A1421C	p.K474T	0	0	-	-	19·521
14	2	13/23	14q11.2	cHet	*SLC7A8*	Solute carrier family 7	c.T1170T	p.Y390Y	-	-	-	--	19·102
1	9	16/23	1q24.3	Het	*MYOC*	Myocilin, trabecular meshwork inducible glucocorticoid response	c.553_555del	p.185_185del	-	-	18·543	-	-
1	1	15/23	1q23.3	Hom	*RXRG*	Retinoid X receptor, gamma	c.118_120del	p.40_40del	-	-	16·973	-	-
2	4	12/23	2p23.1	Het	*CAPN13*	Calpain 13	c.T1787C	p.I596T	0	0	18·074	-	-
2	11	9/23	2q14.3	Het	*MYO7B*	Myosin VIIB	c.A702G	p.Q234Q	0·0006	0	17·660	-	-
4	13	17/23	4q35.2	Hom	*F11*	Coagulation factor XI	c.C453T	p.Y151Y	0	0	16·585	-	-
7	4	26/56	7q22.1	Het	*SERPINE1*	Serpin peptidase inhibitor, clade E, member 1	c.G49A	p.V17I	-	-	16·256	-	-
7	4	31/56	7q36.1	Hom	*KCNH2*	Potassium voltage-gated channel, subfamily H	c.C2900T	:p.P967L	0	0	15·59	-	-
7	6	43/56	7q21.12	Hom	*ABCB1*	ATP-binding cassette, sub-family B, member 1	c.A4006G	p.I1336V	0	0	19·362	-	-

8	9	21/56	8p11.21	Hom	*PLAT*	Plasminogen activator, tissue	c.C1681T	p.R561X	-	-	19·520	-	-
11	1	53/56	11q24.1	Het	*SORL1*	Sortilin-related receptor, L A repeats-containing	c.G237A	p.R79R	0·000096 15	0	20·40	-	-
11	3	19/56	11p15.1	Het	*KCNJ11*	Potassium inwardly-rectifying channel, subfamily J, member 11	c.A828G	p.S276S	-	-	18·636	-	-
12	5	23/56	12q24.21	Het	*TBX3*	T-box 3	c.T1186A	p.S396T	-	-	18·243	-	-
14	8	47/56	14q32.13	Het	*SERPINA1*	Serpin peptidase inhibitor, clade A	c.A1200C	p.E400D	0	0	20·05	-	-
15	2	39/56	15q21.1	Hom	*SLC12A1*	Solute carrier family 12 , member 1	c.A200G	p.Q67R	0	0	19·286	-	-
15	2	29/56	15q21.3	Hom	*LIPC*	Lipase, hepatic	c.G213A	p.T71T	-	-	19·267	-	-
15	2	19/56	15q12	Hom	*ATP10A*	ATPase Phospholipid Transporting 10A	c.A4449G	p.Q1483Q	-	-	15·42	-	-
16	5	28/56	16p11.2	Het	*ALDOA*	Aldolase A, fructose-bisphosphate	c.A151G	p.T51A	0	0	18·556	-	-
19	2	21/56	19p13.2	Het	*LDLR*	Low density lipoprotein receptor	c.G1171A	p.A391T	-	-	17·733	-	-

[£]Exonic. nonsynonymous variants that were considered damaging according to 21 different functional scores from the annotation databases, including SIFT, Polyphen2_HDIV, Polyphen2_HVAR, Likelihood ratio test, MutationTaster, MutationAssessor, FATHMM, fathmm-MKL, RadialSVM, LR, CADD, PROVEAN, MetaSVM and MetaLR, as previously reported.[8] Abbreviations: Hom: Homozygous; Het: heterozygous; cHet: compound heterozygous; #SV: nonsynonymous variants; SNP: Single Nucleotide Polymorphism; ExAC: Exome Aggregation Consortium; AFR: African; EUR: European.

Table S2. Description of the replication study cohort (SCA patients from the Democratic Republic of Congo)

Variables	*Categories*	*Mean +/- SD*	*Value range*	*Number of observations*
Gender	Female/Male (%)	31.8%/69.2%		29
Age (years)		26.1 ± 9.8	18 – 51	29
Hematological indices[1]	Hb (g/dl)	9.1 ± 1.8	5.6 – 14.0	27
	MCV (fl)	9.1 ± 3.1	5.3 – 14.6	27
	WBC (10^9/l)	8.1 ± 3.0	3.0 – 13.6	27
	Platelets (10^9/l)	334 ± 166.5	133 – 737	27
	Neutrophils (10^9/l)	4.6 ± 0.1	2.0 – 10.3	27
Clinical events	Age of diagnosis (years)	6.8 ± 7.1	1 – 39	29
	Vaso-occlusive crisis (No. /year)	1.5 ± 1.2	0 – 5	29
	Stroke	10.3%		3/29
	Leg ulcers	13.8%		4/29
	Hospitalizations (No. /year)	2.1 ± 1.4	0 - 5	29
Treatment	Blood transfusions	31.0%		9/29
	Hydroxyurea (mg/day)	544.1 ± 144	500 – 1000	29
HBB rs334 genotype	HbSS[2]	100%		29/29
HBB haplotype	Bantu/Bantu	50.0%		13/26
	Bantu/Senegal	7.7%		2/26
	Bantu/Benin	7.7%		2/26
	Bantu/Atypical	15.4%		4/26
	Atypical/Atypical	19.2%		5/26
α-globin gene deletion	αα/αα	57.7%		15/26
	αα/α3.7	42.3%		11/26

[1]Hb- Hemoglobin; MCV = mean corpuscular volume; WBCs = white blood cells
[2]HbSS = genotype homozygous for the causal sickle allele HbS

Table S3 | Gene with mutations in SCD patients from Democratic Republic of Congo (DRC)[£].

CHR	Max # SNPs	# Patients	Region	Gene	Gene Name	CDNA Change	Protein Change	EXAc AFR	ExaC EUR
1	11	12/29	1p21.1	*COL11A1*	Collagen Type XI Alpha 1 Chain	c.G4025A	p.G1342D	9·617e-05	0
1	6	9/29	1p36.13	*ALDH4A1*	Aldehyde Dehydrogenase 4 Family Member A1	c.C852A	p.F284L	0	1·505e-05
1	4	17/29	1p36.22	*CLCN6*	Chloride channel 6	c.C2436A	p.F812L	.	.
1	8	12/29	1p36.33	*ATAD3C*	ATPase Family, AAA Domain Containing 3C	c.G425C	p.G142A	0·0014	0
1	1	11/29	1q25.1	*SERPINC1*	Serpin peptidase inhibitor, clade C , member 1	c.G167A	p.R56H	0	0
2	3	11/29	2p23.1	*CAPN13*	Calpain 13	c.G838A	p.A280T	0·9181	0·6884
2	3	23/29	2q14.3	*MYO7B*	Myosin VIIB	c.G5764A	p.G1922R	0	2·609e-05
2	3	19/29	2q22.1	*LRP1B*	LDL Receptor Related Protein 1B	c.A4487G	p.N1496S	0	1·5e-05
2	7	16/29	2q37.3	*COL6A3*	Collagen, type VI, alpha 3	c.A3290G	p.Q1097R	.-	. -
4	9	11/29	4p16.3	*SLC26A1*	Solute Carrier Family 26 Member 1	c.G1060A	p.A354T	0	9·686e-05
4	4	8/29	4q31.3	*LRBA*	LPS Responsive Beige-Like Anchor Protein	c.G3914C	p.R1305P	0	0
7	3	15/29	7q21.11	*HGF*	Hepatocyte growth factor	c.C58A	p.L20I	0·0003	0
7	5	17/29	7q31.33	*GRM8*	Glutamate Metabotropic Receptor 8	c.C1534G	p.P512A	0·0648	0·0001
7	1	21/29	7q36.1	*NOS3*	Nitric oxide synthase 3	c.C1801A	p.Q601K	.-	-.
8	1	11/29	8p21.2	*NKX2-6*	NK2 Homeobox 6	c.G464T	p.R155L	0·0004	0·0001
8	4	13/29	8q24.12	*SNTB1*	Syntrophin, beta 1	c.G19A	p.A7T	0·0278	0
10	6	8/29	10q11.23	*OGDHL*	Oxoglutarate dehydrogenase-like	c.C1259T	p.T420M	0	4·502e-05
11	1	11/29	11q13.3	*IGHMBP2*	Immunoglobulin Mu Binding Protein 2	c.G2857A	p.G953S	0·0003	0

11	3	11/29	11q13.4	*NADSYN1*	NAD synthetase 1	c.A612C	p.Q204H	1	1
12	2	14/29	12p13.33	*TULP3*	Tubby Like Protein 3	c.G254T	p.G85V	0·0003	0
12	1	9/29	12q13.12	*WNT1*	Wnt Family Member 1	c.G620A	p.R207H	0	0
12	2	8/29	12q13.13	*KRT76*	Keratin 76	c.A803T	p.D268V	0·0047	0
12	6	13/29	12q24.31	*OGFOD2*	2-Oxoglutarate And Iron Dependent Oxygenase Domain Containing 2	c.A287G	p.E96G	0	0
16	2	13/29	16p13.3	*CACNA1H*	Calcium channel, voltage-dependent, T type, alpha 1H subunit	c.G6625A	p.A2209T	.	.
16	2	11/29	16q22.1	*PDPR*	Pyruvate Dehydrogenase Phosphatase Regulatory Subunit	c.G2141A	p.R714Q	0	0
16	2	12/29	16q24.3	*SPG7*	SPG7, Paraplegin Matrix AAA Peptidase Subunit	c.C1067T	p.T356M	0	0
17	1	9/29	17p11.2	*ALDH3A1*	Aldehyde Dehydrogenase 3 Family Member A1	c.C623T	p.T208M	0	0
17	3	16/29	17p11.2	*KCNJ12*	Potassium Voltage-Gated Channel Subfamily J Member 12	c.G647A	p.G216D	0·0027	0
17	4	18/29	17p12	*COX10*	COX10, Heme A:Farnesyltransferase Cytochrome C Oxidase Assembly Factor	c.T773A	p.L258H	0·0047	0
17	2	23/29	17q11.2	*MYO1D*	Myosin ID	c.G181T	p.D61Y	0·0002	0
17	7	22/29	17q23.3	*SCN4A*	Sodium Voltage-Gated Channel Alpha Subunit 4	c.C91T	p.R31W	0	3·129e-05
19	3	15/29	19p13.2	*INSR*	Insulin receptor	c.C5G	p.A2G	.-	. -
19	2	11/29	19p13.3	*ABCA7*	ATP Binding Cassette Subfamily A Member 7	c.C191T	p.A64V	0·0001	0
20	1	16/29	20q13.33	*GATA5*	GATA Binding Protein 5	c.G640A	p.G214S	0	0
X	1	8/29	Xq24	*SLC25A5*	Solute Carrier Family 25 Member 5	c.G361T	p.G121C	0·0013	0·0004

[£]Exonic. nonsynonymous variants that were considered damaging according to 21 different functional scores from the annotation databases, including SIFT, LRT, MutationTaster, MutationAssessor, FATHMM, fathmm-MKL, RadialSVM, LR, PROVEAN, MetaSVM, MetaLR, CADD, GERP++, DANN, M-CAP, Eigen, GenoCanyon, Polyphen2 HVAR, Polyphen2 HDIV, PhyloP, and SiPhy, as previously reported.[8] Abbreviations: Max #SNPs: Maximum number of nonsynonymous variant observed among the 3 SCD groups; SNP: Single Nucleotide Polymorphism; ExAC: Exome Aggregation Consortium; AFR: African; EUR: European.

Table S4 | Gene-specific signal of unusual difference in SNPs frequencies between Cameroun Control versus All SCA, "randomly selected", "stroke" and "long survivor" SCD patients.

CHR*	Cytoband	Gene name	P-value from Gene-specific difference in SNPs* frequencies between Cameroun controls versus			
			All SCA patients	SCA random	SCA Stroke	SCA Survivor
1	1p36.13	***CLCNKB***	8.60E-06	0.0177866	0.1132944	0.048514
1	1q23.3	***RXRG***	9.70E-06	0.0691087	0.0083722	0.4035179
1	1p36.13	***CLCNKA***	1.21E-05	0.0120817	0.1510396	0.0304572
11	11p15.4	***HBG2***	1.31E-05	0.0054687	0.4990539	2.32E-01
19	19q13.2	***KCNK6***	1.32E-05	0.7164672	5.77E-02	0.9
1	1q24.1	***FAM78B***	1.42E-05	0.029469	7.96E-02	0.1435747
15	15q24.1	***ULK3***	1.59E-05	0.4647865	0.2752014	1.42E-01
2	2q37.1	***NPPC***	1.90E-05	0.2936289	0.1522729	3.02E-03
17	17p13.2	***GP1BA***	1.93E-05	0.2074716	0.0819473	0.0229895
15	15q26.1	***ASB9P1***	2.02E-05	0.8608001	0.3276578	0.2107018
15	15q24.1	***CSK***	2.49E-05	0.3799891	0.6215994	1.49E-01
3	3p22.2	***CX3CR1***	2.94E-05	0.1352331	1.59E-01	0.0566138
2	2q12.1	***FHL2***	3.21E-05	0.2365149	0.0794517	0.2868306
17	17q21.31	***WNK4***	3.40E-05	0.4074042	7.53E-03	0.0944508
10	10q24.31	***CHUK***	3.54E-05	0.2827386	0.4938868	2.97E-01
7	7q31.2	***CAV1***	3.59E-05	0.0969133	0.0739469	0.0280981
11	11p11.2	***F2***	3.72E-05	0.1804408	0.2288114	0.3126277
1	1q24.2	***SLC19A2***	3.85E-05	0.28671	0.0408285	0.0239266
16	16p13.3	***DNASE1***	3.99E-05	0.1121478	7.64E-03	0.1996761
9	9q21.32	***UBQLN1***	4.16E-05	0.0051538	6.65E-01	0.9
17	17q25.1	***LLGL2***	4.56E-05	0.0237748	4.11E-02	0.5014317
11	11p15.4	***HBB***	4.57E-05	0.02027	7.14E-02	0.080082
4	4q31.21	***GYPA***	4.73E-05	0.1808737	0.063652	0.5032097
11	11p15.1	***SAA1***	4.75E-05	0.054711	7.75E-01	0.2582273

4	4q35.2	***F11***	4.77E-05	0.1667932	0.0381918	0.1083799
5	5q15	***ERAP1***	4.79E-05	0.0398614	0.0467189	8.73E-02
12	12q24.31	***P2RX4***	4.99E-05	0.047334	3.17E-02	0.186381
8	8p11.21	***PLAT***	0.018	0.157	1.80E-02	0.027
2	2q32.2	***PMS1***	3.40E-02	0.074	0.23	0.864
19	19q13.32	***APOE***	6.15E-02	0.1367854	0.5361577	3.32E-05
16	16p13.11	***NTAN1***	6.47E-02	0.1190184	0.8327524	1.08E-05
9	9q34.3	***FUT7***	6.85E-02	0.0144899	0.0527418	1.14E-05
17	17p13.2	***CTNS***	0.0808225	0.2485278	4.10E-05	2.49E-01
1	1q24.2	***F5***	9.47E-02	0.411426	0.1042254	4.33E-05
1	1q31.3	***CFH***	0.1126248	0.1202527	3.65E-05	0.2054329
20	20p13	***PRNP***	0.1182381	0.2255724	0.3606476	4.19E-05
11	11q13.4	***P2RY2***	1.36E-01	0.4513712	0.0314428	4.39E-05
5	5q31.3	***NR3C1***	1.46E-01	0.5880721	4.89E-05	0.2001723
16	16q23.1	***MON1B***	1.48E-01	0.2350079	0.3612341	4.89E-05
5	5q23.1	***SEMA6A***	0.1506902	0.0952938	4.35E-05	3.62E-01
17	17q21.2	***CNP***	0.1588079	0.4227699	0.5361959	4.71E-05
1	1p34.2	***EDN2***	1.67E-01	0.4813942	4.58E-05	0.5188978
1	1q25.1	***SERPINC1***	1.72E-01	0.2501583	0.9	4.75E-05
22	22q11.23	***ADORA2A***	0.1834713	0.6887537	0.5334695	4.82E-05
1	1p35.2	***FABP3***	0.1836854	0.3136781	7.78E-01	4.04E-05
14	14q22.2	***GCH1***	0.2018993	0.3364537	0.6292693	4.15E-05
5	5q31.1	***IL4***	0.2282411	0.1718836	4.66E-05	0.9
3	3q25.33	***IL12A***	0.2372261	0.3222783	3.74E-05	4.72E-01
5	5q31.1	***IL13***	0.245173	0.1548332	2.49E-05	9.00E-01
12	12p13.31	***GNB3***	0.2529551	0.7882165	3.57E-05	0.5258433
22	22q11.23	***GSTT1***	0.2960057	0.2141566	1.40E-06	0.4283995
19	19q13.2	***SPTBN4***	0.3233646	0.9	3.54E-05	0.3603377
6	6q14.2	***PRSS35***	0.3480714	0.2432587	4.30E-06	0.1925617
9	9q22.31	***ROR2***	0.388616	0.434711	4.64E-05	0.6851996
2	2p25.1	***HPCAL1***	0.4900588	0.8756077	0.4823774	3.76E-05

7	7q21.11	***HGF***	0.00498	0.9	0.0236	0.0391
1	1q23.3	***FCGR2A***	0.05173812	0.9	0.4546349	1.77E-05
3	3p21.31	***CCR3***	0.527461	0.8870905	0.729255	3.24E-05
16	16p13.3	***CACNA1H***	0.00185	0.0748	0.0558	0.0521
2	2q32.1	***CALCRL***	0.7161659	0.5468703	2.35E-05	0.9
17	17q21.31	***WNT3***	0.7387889	0.1083799	3.45E-05	0.4606751
7	7q36.3	***VIPR2***	0.8171543	0.6129953	4.30E-05	0.2421205
9	9p13.3	***NPR2***	0.8628314	0.2551507	6.90E-06	0.4430517
11	11q13.4	***NADSYN1***	0.009	0.0374	0.04	0.0245

.*Abbreviations: CHR: chromosome; SNPs: Single Nucleotide Polymorphism.

Table S5. Details of mutations identified within genes (Table S1) in SCA patients from Cameroon									
**#CHROM**	**POS**	**SNP**	**A1/A2**	**avsnp147**	**Gene.refGene**	**cDNA_ Change**	**Protein Change**	**AAChange.refGene**	**Siphy Score**
**1**	**11876692**	**rs57044879**	**G/A**	**rs57044879**	***CLCN6***	**1p36.22**	**exon4:c.G234A**	**exon4:p.A78A**	**16.37**
1	11884586	rs60602304	G/A	rs60602304	*CLCN6*	1p36.22	exon8:c.G624A	exon8:p.S208S	13.18
1	11888618	chr1:11888618	G/C	chr1:11888618	*CLCN6*	1p36.22	exon12:c.G1058C	exon12:p.C353S	13.99
1	11894062	rs147341529	C/T	rs147341529	*CLCN6*	1p36.22	exon15:c.C1501T	exon15:p.R501C	13.20
1	11897409	chr1:11897409	C/A	chr1:11897409	*CLCN6*	1p36.22	exon20:c.C2148A	exon20:p.P716P	13.01
**1**	**165386410**	**chr1:165386410**	**TGAA/T**	**chr1:165386410**	***RXRG***	**1q23.3**	**exon5:c.118_120del**	**exon5:p.40_40del**	**13.82**
1	169505792	rs116809837	G/A	rs116809837	*F5*	1q24.2	exon14:c.C4923T	exon14:p.L1641L	13.13
1	169510118	rs9332608	G/A	rs9332608	*F5*	1q24.2	exon13:c.C4210T	exon13:p.P1404S	13.24
1	169510233	rs9332607	G/A	rs9332607	*F5*	1q24.2	exon13:c.C4095T	exon13:p.T1365T	13.24
1	169510380	rs9287090	G/A	rs9287090	*F5*	1q24.2	exon13:c.C3948T	exon13:p.L1316L	12.92
1	169510475	rs1046712	G/T	rs1046712	*F5*	1q24.2	exon13:c.C3853A	exon13:p.L1285I	12.22
1	169510524	rs1800594	A/G	rs1800594	*F5*	1q24.2	exon13:c.T3804C	exon13:p.S1268S	12.21
1	169510890	rs6005	G/C	rs6005	*F5*	1q24.2	exon13:c.C3438G	exon13:p.H1146Q	12.29
1	169511166	rs149026031	T/G	rs149026031	*F5*	1q24.2	exon13:c.A3162C	exon13:p.E1054D	12.15
1	169511389	rs9332605	C/A	rs9332605	*F5*	1q24.2	exon13:c.G2939T	exon13:p.R980L	12.16
1	169511445	rs144026312	A/G	rs144026312	*F5*	1q24.2	exon13:c.T2883C	exon13:p.D961D	12.32
1	169511903	rs6031	G/A	rs6031	*F5*	1q24.2	exon13:c.C2425T	exon13:p.P809S	12.34
**1**	**169512078**	**rs377011882**	**CTCT/C**	**rs377011882**	***F5***	**1q24.2**	**exon13:c.2247_2249 del**	**exon13:p.749_750 del**	**18.04**
1	169512093	rs6017	A/G	rs6017	*F5*	1q24.2	exon13:c.T2235C	exon13:p.N745N	12.35
1	169512120	rs6016	G/A	rs6016	*F5*	1q24.2	exon13:c.C2208T	exon13:p.I736I	13.46

1	169512199	rs115954845	T/C	rs115954845	*F5*	1q24.2	exon13:c.A2129G	exon13:p.H710R	14.07
1	169512223	rs78958618	G/A	rs78958618	*F5*	1q24.2	exon13:c.C2105T	exon13:p.T702I	13.38
1	169526020	rs9332578	G/A	rs9332578	*F5*	1q24.2	exon6:c.C816T	exon6:p.N272N	15.29
1	169529826	rs6022	A/C	rs6022	*F5*	1q24.2	exon4:c.G552G	exon4:p.S184S	12.40
1	169529973	rs6029	T/C	rs6029	*F5*	1q24.2	exon4:c.G405G	exon4:p.A135A	14.01
1	171605081	rs145977437	T/C	rs145977437	*MYOC*	1q24.3	exon3:c.A1499G	exon3:p.K500R	12.42
1	171605392	rs61730975	C/T	rs61730975	*MYOC*	1q24.3	exon3:c.G1188A	exon3:p.E396E	16.00
1	171605526	rs61745146	C/T	rs61745146	*MYOC*	1q24.3	exon3:c.G1054A	exon3:p.E352K	12.44
1	171605595	rs146391864	C/.	rs146391864	*MYOC*	1q24.3	exon3:c.G985G	exon3:p.V329V	12.85
1	171605605	rs61730976	C/T	rs61730976	*MYOC*	1q24.3	exon3:c.G975A	exon3:p.T325T	12.76
1	171621191	chr1:171621191	C/A	chr1:171621191	*MYOC*	1q24.3	exon1:c.G561T	exon1:p.Q187H	13.47
**1**	**171621196**	**chr1:171621196**	**GACA/G**	**chr1:171621196**	***MYOC***	**1q24.3**	**exon1:c.553_555del**	**exon1:p.185_185del**	**16.18**
1	171621275	rs61730977	T/C	rs61730977	*MYOC*	1q24.3	exon1:c.A477G	exon1:p.L159L	14.49
1	171621713	rs12082573	A/C	rs12082573	*MYOC*	1q24.3	exon1:c.T39G	exon1:p.P13P	13.85
**1**	**173878832**	**rs5878**	**C/T**	**rs5878**	***SERPINC1***	**1q25.1**	**exon5:c.A1011A**	**exon5:p.Q337Q**	**15.11**
1	173878862	rs5877	C/T	rs5877	*SERPINC1*	1q25.1	exon5:c.A981A	exon5:p.V327V	13.92
1	173878985	rs139463995	C/G	rs139463995	*SERPINC1*	1q25.1	exon5:c.G858C	exon5:p.Q286H	14.53
1	173881122	rs2227606	T/C	rs2227606	*SERPINC1*	1q25.1	exon3:c.A439G	exon3:p.T147A	14.04
1	173883886	chr1:173883886	C/A	chr1:173883886	*SERPINC1*	1q25.1	exon2:c.G213T	exon2:p.K71N	15.05
**1**	**203652444**	**rs1419114**	**G/A**	**rs1419114**	***ATP2B4***	**1q32.1**	**exon2:c.A111A**	**exon2:p.S37S**	**15.86**
1	203667409	rs2228445	C/T	rs2228445	*ATP2B4*	1q32.1	exon3:c.T318T	exon3:p.L106L	13.57
1	203672867	rs145963279	T/C	rs145963279	*ATP2B4*	1q32.1	exon8:c.T1025C	exon8:p.V342A	12.58
1	203676326	chr1:203676326	C/A	chr1:203676326	*ATP2B4*	1q32.1	exon9:c.C1289A	exon9:p.T430N	14.59
1	203676332	chr1:203676332	C/A	chr1:203676332	*ATP2B4*	1q32.1	exon9:c.C1295A	exon9:p.S432X	12.60
1	203677220	rs114362667	C/T	rs114362667	*ATP2B4*	1q32.1	exon10:c.C1545T	exon10:p.T515T	14.01

1	203678536	rs74402274	T/C	rs74402274	*ATP2B4*	1q32.1	exon11:c.T1665C	exon11:p.N555N	13.62
1	203680173	rs75360548	T/C	rs75360548	*ATP2B4*	1q32.1	exon12:c.T1968C	exon12:p.N656N	12.03
1	203681255	rs2229565	C/T	rs2229565	*ATP2B4*	1q32.1	exon13:c.C2199T	exon13:p.N733N	12.24
**2**	**30957326**	**rs2276568**	**A/G**	**rs2276568**	***CAPN13***	**2p23.1**	**exon19:c.T1787C**	**exon19:p.I596T**	**15.65**
2	30964792	rs75691612	G/C	rs75691612	*CAPN13*	2p23.1	exon15:c.C1518G	exon15:p.F506L	12.66
2	30966387	rs113891539	C/A	rs113891539	*CAPN13*	2p23.1	exon13:c.G1307T	exon13:p.S436I	13.67
2	31000437	rs150868423	G/A	rs150868423	*CAPN13*	2p23.1	exon3:c.C267T	exon3:p.G89G	12.68
2	74454158	rs146665416	G/C	rs146665416	*SLC4A5*	2p13.1	exon27:c.C3016G	exon27:p.L1006V	12.09
**2**	**74458420**	**rs114300772**	**A/A**	**rs114300772**	***SLC4A5***	**2p13.1**	**exon25:c.C2790T**	**exon25:p.T930T**	**20.28**
2	74460685	rs3796109	C/T	rs3796109	*SLC4A5*	2p13.1	exon23:c.G2439A	exon23:p.T813T	14.71
2	74466594	rs4853018	G/A	rs4853018	*SLC4A5*	2p13.1	exon21:c.C2187T	exon21:p.G729G	12.02
2	74479413	chr2:74479413	GCCA/G	rs764953142	*SLC4A5*	2p13.1	exon16:c.1368_1370 del	exon16:p.456_457 del	14.73
2	74489323	rs17009792	C/T	rs17009792	*SLC4A5*	2p13.1	exon11:c.G752A	exon11:p.S251N	12.74
2	74492388	chr2:74492388	C/A	chr2:74492388	*SLC4A5*	2p13.1	exon9:c.G405T	exon9:p.W135C	17.75
**2**	**128331604**	**rs193069171**	**A/G**	**rs193069171**	***MYO7B***	**2q14.3**	**exon7:c.A702G**	**exon7:p.Q234Q**	**14.66**
2	128351163	rs147949489	G/A	rs147949489	*MYO7B*	2q14.3	exon18:c.G2188A	exon18:p.V730M	12.77
2	128351183	rs115592021	A/C	rs115592021	*MYO7B*	2q14.3	exon18:c.A2208C	exon18:p.K736N	12.78
2	128364873	rs199821381	G/A	rs199821381	*MYO7B*	2q14.3	exon21:c.G2517A	exon21:p.L839L	13.79
2	128367092	rs777432	G/A	rs777432	*MYO7B*	2q14.3	exon23:c.G2826A	exon23:p.S942S	13.02
2	128367144	rs200386846	G/A	rs200386846	*MYO7B*	2q14.3	exon23:c.G2878A	exon23:p.E960K	12.81
2	128367433	rs61741454	G/A	rs61741454	*MYO7B*	2q14.3	exon24:c.G3034A	exon24:p.V1012I	12.85
2	128381717	rs2245408	G/A	rs2245408	*MYO7B*	2q14.3	exon29:c.G3791A	exon29:p.R1264Q	12.53
2	128381773	rs116176015	A/G	rs116176015	*MYO7B*	2q14.3	exon29:c.A3847G	exon29:p.I1283V	12.84
2	128381808	rs13422424	C/T	rs13422424	*MYO7B*	2q14.3	exon29:c.C3882T	exon29:p.H1294H	12.80
2	128381861	rs61743523	G/A	rs61743523	*MYO7B*	2q14.3	exon29:c.G3935A	exon29:p.R1312Q	12.96

2	128381889	rs61745600	C/T	rs61745600	*MYO7B*	2q14.3	exon29:c.C3963T	exon29:p.F1321F	12.87
2	128385982	rs147310604	C/T	rs147310604	*MYO7B*	2q14.3	exon33:c.C4418T	exon33:p.T1473M	13.08
2	128386040	rs376979966	G/A	rs376979966	*MYO7B*	2q14.3	exon33:c.G4476A	exon33:p.V1492V	12.89
2	128388790	rs13025791	G/A	rs13025791	*MYO7B*	2q14.3	exon36:c.G4869A	exon36:p.E1623E	12.80
2	128388794	rs142758251	G/A	rs142758251	*MYO7B*	2q14.3	exon36:c.G4873A	exon36:p.D1625N	12.91
2	128388862	rs13025959	G/C	rs13025959	*MYO7B*	2q14.3	exon36:c.G4941C	exon36:p.E1647D	12.90
2	128388902	rs201972665	G/A	rs201972665	*MYO7B*	2q14.3	exon36:c.G4981A	exon36:p.V1661I	12.93
2	128388919	chr2:128388919	C/A	chr2:128388919	*MYO7B*	2q14.3	exon36:c.C4998A	exon36:p.I1666I	12.74
2	128389939	chr2:128389939	GC/.	rs746320826	*MYO7B*	2q14.3	exon38:c.5291delC	exon38:p.A1764fs	12.65
2	128389950	chr2:128389950	CAG/.	chr2:128389950	*MYO7B*	2q14.3	exon38:c.5303delG	exon38:p.S1768fs	12.96
2	128390878	rs187045307	C/T	rs187045307	*MYO7B*	2q14.3	exon39:c.C5373T	exon39:p.A1791A	12.77
2	128390935	rs146807651	T/C	rs146807651	*MYO7B*	2q14.3	exon39:c.T5430C	exon39:p.S1810S	12.88
2	128392173	rs61738660	C/T	rs61738660	*MYO7B*	2q14.3	exon41:c.C5550T	exon41:p.V1850V	12.99
2	128393359	rs150007517	G/A	rs150007517	*MYO7B*	2q14.3	exon43:c.G5805A	exon43:p.A1935A	12.60
2	128394955	rs11686946	A/G	rs11686946	*MYO7B*	2q14.3	exon47:c.A6314G	exon47:p.Q2105R	12.70
**2**	**190660503**	**rs5742980**	**T/C**	**rs5742980**	***PMS1***	**2q32.2**	**exon4:c.T141C**	**exon4:p.Y47Y**	**20.02**
2	190670407	rs2066457	T/C	rs2066457	*PMS1*	2q32.2	exon5:c.T345C	exon5:p.D115D	14.98
2	190671168	chr2:190671168	G/A	chr2:190671168	*PMS1*	2q32.2	exon5:c.G438A	exon5:p.Q146Q	13.10
2	190719742	rs74512161	G/A	rs74512161	*PMS1*	2q32.2	exon10:c.G1744A	exon10:p.V582I	13.10
2	190728779	rs61736573	G/A	rs61736573	*PMS1*	2q32.2	exon11:c.G2167A	exon11:p.E723K	14.89
2	190732599	rs55859858	C/G	rs55859858	*PMS1*	2q32.2	exon12:c.C2417G	exon12:p.T806S	15.07
2	190742119	rs147566508	G/A	rs147566508	*PMS1*	2q32.2	exon14:c.G2756A	exon14:p.R919H	14.08
2	211421452	rs3835047	ATCT/A	rs3835047	*CPS1*	2q34	exon2:c.14_16del	exon2:p.5_6del	15.10
2	211456639	rs2229589	T/C	rs2229589	*CPS1*	2q34	exon11:c.C1050C	exon11:p.T350T	12.54
**2**	**211456675**	**rs34022862**	**G/G**	**rs34022862**	***CPS1***	**2q34**	**exon11:c.C1086G**	**exon11:p.V362V**	**19.27**
2	211473157	rs41272667	C/T	rs41272667	*CPS1*	2q34	exon20:c.C2283T	exon20:p.S761S	14.11

2	211476843	rs35678745	C/A	rs35678745	*CPS1*	2q34	exon21:c.C2412A	exon21:p.V804V	13.13
2	211481257	rs2287599	G/C	rs2287599	*CPS1*	2q34	exon22:c.C2697C	exon22:p.G899G	15.14
2	211507277	rs79627159	C/T	rs79627159	*CPS1*	2q34	exon26:c.C3047T	exon26:p.T1016M	14.12
2	211507281	rs35374255	G/C	rs35374255	*CPS1*	2q34	exon26:c.G3051C	exon26:p.V1017V	13.16
2	211513215	rs76340296	G/A	rs76340296	*CPS1*	2q34	exon28:c.G3373A	exon28:p.A1125T	15.17
2	211540507	rs1047891	C/A	rs1047891	*CPS1*	2q34	exon37:c.C4235A	exon37:p.T1412N	12.18
2	211540550	rs138395129	C/G	rs138395129	*CPS1*	2q34	exon37:c.C4278G	exon37:p.L1426L	14.19
**2**	**238283288**	**rs36062562**	**C/T**	**rs36062562**	***COL6A3***	**2q37.3**	**exon8:c.G3446A**	**exon8:p.R1149Q**	**14.20**
2	238283544	rs369810455	G/A	rs369810455	*COL6A3*	2q37.3	exon8:c.C3190T	exon8:p.R1064W	12.31
2	238285431	rs34367758	G/A	rs34367758	*COL6A3*	2q37.3	exon7:c.C3054T	exon7:p.N1018N	13.12
2	238287800	rs36092870	C/T	rs36092870	*COL6A3*	2q37.3	exon6:c.G1976A	exon6:p.R659H	13.12
2	238289664	rs76576170	G/A	rs76576170	*COL6A3*	2q37.3	exon5:c.C1791T	exon5:p.F597F	13.12
2	238289669	rs34934127	C/A	rs34934127	*COL6A3*	2q37.3	exon5:c.G1786T	exon5:p.A596S	12.15
2	238289817	rs112040282	G/A	rs112040282	*COL6A3*	2q37.3	exon5:c.C1638T	exon5:p.A546A	12.26
2	238296323	rs114549120	A/G	rs114549120	*COL6A3*	2q37.3	exon4:c.T1214C	exon4:p.F405S	12.47
2	238296655	rs7561625	G/A	rs7561625	*COL6A3*	2q37.3	exon4:c.C882T	exon4:p.F294F	12.28
4	187195373	rs5973	C/T	rs5973	*F11*	4q35.2	exon5:c.C429T	exon5:p.D143D	12.29
**4**	**187195397**	**rs34807019**	**C/T**	**rs34807019**	***F11***	**4q35.2**	**exon5:c.C453T**	**exon5:p.Y151Y**	**15.30**
4	187201211	rs5974	A/G	rs5974	*F11*	4q35.2	exon8:c.A801G	exon8:p.T267T	12.31
4	187205301	rs5970	T/C	rs5970	*F11*	4q35.2	exon11:c.T1191C	exon11:p.G397G	12.42
4	187208968	rs5975	C/T	rs5975	*F11*	4q35.2	exon14:c.C1707T	exon14:p.D569D	12.33
4	187209702	rs5971	G/T	rs5971	*F11*	4q35.2	exon15:c.G1812T	exon15:p.R604R	13.04
4	187209729	rs5976	G/A	rs5976	*F11*	4q35.2	exon15:c.G1839A	exon15:p.E613E	12.35
**5**	**7870973**	**rs1801394**	**A/G**	**rs1801394**	***MTRR***	**5p15.31**	**exon2:c.A147G**	**exon2:p.I49M**	**15.16**
5	7873500	rs138612190	C/T	rs138612190	*MTRR*	5p15.31	exon3:c.C225T	exon3:p.T75T	12.37
5	70888760	rs144578800	A/G	rs144578800	*MCCC2*	5q13.2	exon2:c.A137G	exon2:p.Y46C	13.08

**5**	**70931043**	**rs112793062**	**T/C**	**rs112793062**	***MCCC2***	**5q13.2**	**exon10:c.T969C**	**exon10:p.A323A**	**17.39**
5	70945075	rs10064079	G/A	rs10064079	*MCCC2*	5q13.2	exon14:c.A1368A	exon14:p.A456A	15.14
5	131705723	rs144020613	T/A	rs144020613	*SLC22A5*	5q31.1	exon1:c.T59A	exon1:p.L20H	12.14
**5**	**131705941**	**chr5:131705941**	**TCGG/.**	**chr5:131705941**	***SLC22A5***	**5q31.1**	**exon1:c.280delG**	**exon1:p.A94fs**	**18.12**
5	131705949	rs2631365	T/C	rs2631365	*SLC22A5*	5q31.1	exon1:c.T285C	exon1:p.L95L	12.43
5	131721174	rs274558	A/G	rs274558	*SLC22A5*	5q31.1	exon5:c.A879G	exon5:p.L293L	13.44
5	131726578	rs139775414	A/G	rs139775414	*SLC22A5*	5q31.1	exon8:c.A1321G	exon8:p.M441V	12.14
5	131728225	rs142355575	A/G	rs142355575	*SLC22A5*	5q31.1	exon9:c.A1440G	exon9:p.T480T	12.46
5	131729880	rs148233131	G/T	rs148233131	*SLC22A5*	5q31.1	exon11:c.G1662T	exon11:p.M554I	13.07
5	131729935	rs11568525	C/T	rs11568525	*SLC22A5*	5q31.1	exon11:c.C1717T	exon11:p.P573S	14.48
6	7727271	rs111588693	G/A	rs111588693	*BMP6*	6p24.3	exon1:c.G83A	exon1:p.R28Q	16.09
6	7727546	chr6:7727546	C/A	chr6:7727546	*BMP6*	6p24.3	exon1:c.C358A	exon1:p.P120T	12.50
6	7727590	chr6:7727590	C/A	chr6:7727590	*BMP6*	6p24.3	exon1:c.C402A	exon1:p.L134L	12.56
**6**	**7727753**	**chr6:7727753**	**GC/.**	**chr6:7727753**	***BMP6***	**6p24.3**	**exon1:c.566delC**	**exon1:p.A189fs**	**16.72**
6	7727824	rs199789965	G/.	rs199789965	*BMP6*	6p24.3	exon1:c.G636G	exon1:p.A212A	12.15
6	7727849	chr6:7727849	C/.	chr6:7727849	*BMP6*	6p24.3	exon1:c.C661C	exon1:p.L221L	12.34
6	7845478	rs148916269	G/A	rs148916269	*BMP6*	6p24.3	exon2:c.G770A	exon2:p.R257H	12.55
6	7862541	rs61733612	C/T	rs61733612	*BMP6*	6p24.3	exon4:c.C1014T	exon4:p.H338H	12.86
6	7862589	rs17558	C/T	rs17558	*BMP6*	6p24.3	exon4:c.C1062T	exon4:p.D354D	12.67
6	7862631	rs17557	C/G	rs17557	*BMP6*	6p24.3	exon4:c.G1104G	exon4:p.V368V	12.58
6	7880291	rs150397946	A/.	rs150397946	*BMP6*	6p24.3	exon6:c.A1349A	exon6:p.N450N	12.99
6	7880326	chr6:7880326	C/A	chr6:7880326	*BMP6*	6p24.3	exon6:c.C1384A	exon6:p.Q462K	12.16
6	7880519	rs149391648	C/G	rs149391648	*BMP6*	6p24.3	exon7:c.C1485G	exon7:p.S495S	12.78
**6**	**133004281**	**rs61729583**	**A/G**	**rs61729583**	***VNN1***	**6q23.2**	**exon7:c.T1540C**	**exon7:p.X514Q**	**15.12**
**7**	**81374351**	**rs5745666**	**A/G**	**rs5745666**	***HGF***	**7q21.11**	**exon6:c.T696C**	**exon6:p.H232H**	**14.93**
**7**	**87133606**	**chr7:87133606**	**T/C**	**chr7:87133606**	***ABCB1***	**7q21.12**	**exon32:c.A4006G**	**exon32:p.I1336V**	**15.64**

7	87138645	rs1045642	G/A	rs1045642	*ABCB1*	7q21.12	exon30:c.T3645T	exon30:p.I1215I	12.16
7	87138659	rs2229107	A/T	rs2229107	*ABCB1*	7q21.12	exon30:c.T3631A	exon30:p.S1211T	12.66
7	87144678	rs28401798	G/C	rs28401798	*ABCB1*	7q21.12	exon29:c.C3361G	exon29:p.P1121A	13.67
7	87174198	rs35023033	G/A	rs35023033	*ABCB1*	7q21.12	exon20:c.C2215T	exon20:p.R739C	12.98
7	87179601	rs1128503	G/A	rs1128503	*ABCB1*	7q21.12	exon16:c.T1446T	exon16:p.G482G	12.69
**7**	**100771723**	**rs6090**	**G/A**	**rs6090**	***SERPINE1***	**7q22.1**	**exon2:c.G49A**	**exon2:p.V17I**	**14.70**
7	100771765	rs141347752	G/A	rs141347752	*SERPINE1*	7q22.1	exon2:c.G91A	exon2:p.V31M	12.71
7	100775298	rs2227670	C/T	rs2227670	*SERPINE1*	7q22.1	exon4:c.C648T	exon4:p.D216D	13.12
**7**	**150644759**	**rs199473016**	**G/A**	**rs199473016**	***KCNH2***	**7q36.1**	**exon12:c.C2900T**	**exon12:p.P967L**	**16.13**
7	150644930	rs199473436	G/A	rs199473436	*KCNH2*	7q36.1	exon12:c.C2729T	exon12:p.P910L	13.17
7	150647022	rs370393086	G/A	rs370393086	*KCNH2*	7q36.1	exon9:c.C2632T	exon9:p.R878C	12.75
7	150647150	chr7:150647150	AG/A	rs546898924	*KCNH2*	7q36.1	exon9:c.2503delC	exon9:p.L835fs	13.76
7	150655505	rs139533994	G/A	rs139533994	*KCNH2*	7q36.1	exon4:c.C558T	exon4:p.G186G	12.77
7	150693556	rs3918166	G/A	rs3918166	*NOS3*	7q36.1	exon4:c.G335A	exon4:p.R112Q	13.88
**7**	**150693561**	**chr7:150693561**	**TCCCCC GGC/T**	**chr7:150693561**	***NOS3***	**7q36.1**	**exon4:c.341_348del**	**exon4:p.S114fs**	**22.79**
7	150704250	rs2566514	G/C	rs2566514	*NOS3*	7q36.1	exon17:c.C1998C	exon17:p.A666A	12.18
7	150704310	chr7:150704310	C/T	rs779245100	*NOS3*	7q36.1	exon17:c.C2058T	exon17:p.D686D	13.18
7	150707312	rs34967063	G/A	rs34967063	*NOS3*	7q36.1	exon21:c.G2622A	exon21:p.L874L	15.82
7	150707344	rs3918201	G/T	rs3918201	*NOS3*	7q36.1	exon21:c.G2654T	exon21:p.R885M	13.83
7	150710392	rs3730011	G/A	rs3730011	*NOS3*	7q36.1	exon25:c.G3180A	exon25:p.E1060E	12.84
7	150710461	chr7:150710461	C/A	chr7:150710461	*NOS3*	7q36.1	exon25:c.C3249A	exon25:p.N1083K	14.85
7	150710907	rs3918211	T/C	rs3918211	*NOS3*	7q36.1	exon26:c.T3351C	exon26:p.V1117V	13.09
**8**	**42033519**	**rs1804182**	**G/A**	**rs1804182**	***PLAT***	**8p11.21**	**exon14:c.C1681T**	**exon14:p.R561X**	**15.10**
8	42036466	rs62001886	A/T	rs62001886	*PLAT*	8p11.21	exon13:c.T1479A	exon13:p.A493A	12.88
8	42036577	rs1136159	A/G	rs1136159	*PLAT*	8p11.21	exon13:c.T1368C	exon13:p.S456S	13.89

8	42038169	chr8:42038169	C/A	chr8:42038169	*PLAT*	8p11.21	exon10:c.G924T	exon10:p.Q308H	12.90
8	42039483	rs8178777	C/T	rs8178777	*PLAT*	8p11.21	exon9:c.G861A	exon9:p.T287T	14.91
8	42042600	chr8:42042600	C/A	chr8:42042600	*PLAT*	8p11.21	exon7:c.G630T	exon7:p.E210D	13.92
8	42044954	rs1058720	A/G	rs1058720	*PLAT*	8p11.21	exon6:c.C501C	exon6:p.D167D	12.93
**8**	**121554236**	**chr8:121554236**	**G/A**	**chr8:121554236**	***SNTB1***	**8q24.12**	**exon6:c.C1338T**	**exon6:p.C446C**	**14.14**
8	121587349	rs116157159	G/A	rs116157159	*SNTB1*	8q24.12	exon4:c.C1113T	exon4:p.H371H	12.95
8	121824054	chr8:121824054	A/AGCC	rs547154887	*SNTB1*	8q24.12	exon1:c.29_30insGGC	exon1:p.A10delinsAA	13.06
8	121824063	rs190526297	C/A	rs190526297	*SNTB1*	8q24.12	exon1:c.G21T	exon1:p.A7A	12.97
10	8100578	chr10:8100578	C/G	chr10:8100578	*GATA3*	10p14	exon3:c.C552G	exon3:p.L184L	13.98
**10**	**8100632**	**rs2228254**	**T/C**	**rs2228254**	***GATA3***	**10p14**	**exon3:c.T606C**	**exon3:p.R202R**	**20.99**
**10**	**50943376**	**rs113014306**	**C/T**	**rs113014306**	***OGDHL***	**10q11.23**	**exon23:c.G2931A**	**exon23:p.A977A**	**13.00**
10	50950976	rs11101224	G/A	rs11101224	*OGDHL*	10q11.23	exon15:c.C1910T	exon15:p.T637M	12.20
10	50951018	rs34877195	G/C	rs34877195	*OGDHL*	10q11.23	exon15:c.C1868G	exon15:p.S623C	12.22
10	50954850	rs75974530	G/A	rs75974530	*OGDHL*	10q11.23	exon10:c.C1242T	exon10:p.S414S	13.09
10	50964932	rs140510079	G/A	rs140510079	*OGDHL*	10q11.23	exon3:c.C265T	exon3:p.R89W	13.01
**11**	**17408550**	**rs5214**	**T/C**	**rs5214**	***KCNJ11***	**11p15.1**	**exon2:c.A828G**	**exon2:p.S276S**	**13.77**
11	17408630	rs5215	T/C	rs5215	*KCNJ11*	11p15.1	exon2:c.G748G	exon2:p.V250V	12.64
11	17409531	rs112070496	C/T	rs112070496	*KCNJ11*	11p15.1	exon1:c.G108A	exon1:p.V36V	12.71
**11**	**31814879**	**rs3026384**	**G/A**	**rs3026384**	***PAX6***	**11p13**	**exon9:c.C1139T**	**exon9:p.S380L**	**22.08**
11	31824263	rs141873759	G/T	rs141873759	*PAX6*	11p13	exon5:c.C130A	exon5:p.R44R	12.33
11	71164364	rs148242268	G/A	rs148242268	*NADSYN1*	11q13.4	exon1:c.G22A	exon1:p.A8T	12.21
11	71169547	rs2276360	G/C	rs2276360	*NADSYN1*	11q13.4	exon3:c.G220C	exon3:p.V74L	12.11
**11**	**71169583**	**rs35588716**	**A/A**	**rs35588716**	***NADSYN1***	**11q13.4**	**exon3:c.G256A**	**exon3:p.V86M**	**17.12**
11	71185443	rs145980605	C/T	rs145980605	*NADSYN1*	11q13.4	exon9:c.C669T	exon9:p.N223N	12.13
11	71185479	rs2276354	C/T	rs2276354	*NADSYN1*	11q13.4	exon9:c.T705T	exon9:p.C235C	12.13

11	71185504	rs371173837	G/A	rs371173837	*NADSYN1*	11q13.4	exon9:c.G730A	exon9:p.G244S	12.31
11	71185518	rs2186778	C/T	rs2186778	*NADSYN1*	11q13.4	exon9:c.T744T	exon9:p.I248I	12.14
11	71191851	rs76770512	G/C	rs76770512	*NADSYN1*	11q13.4	exon11:c.G924C	exon11:p.S308S	12.17
11	71192439	rs59379414	C/A	rs59379414	*NADSYN1*	11q13.4	exon12:c.C1036A	exon12:p.R346R	12.18
11	71195379	rs116695422	C/T	rs116695422	*NADSYN1*	11q13.4	exon15:c.C1341T	exon15:p.I447I	12.21
11	71209518	chr11:71209518	C/T	rs768597235	*NADSYN1*	11q13.4	exon20:c.C2014T	exon20:p.R672X	12.22
11	71212387	rs12282060	G/A	rs12282060	*NADSYN1*	11q13.4	exon21:c.G2110A	exon21:p.G704S	12.67
**11**	**121323277**	**rs114331262**	**G/A**	**rs114331262**	***SORL1***	**11q24.1**	**exon1:c.G237A**	**exon1:p.R79R**	**14.42**
**12**	**115112554**	**rs78115331**	**A/T**	**rs78115331**	***TBX3***	**12q24.21**	**exon7:c.T1186A**	**exon7:p.S396T**	**14.31**
12	115112586	rs141004177	T/C	rs141004177	*TBX3*	12q24.21	exon7:c.A1154G	exon7:p.H385R	12.22
12	115112600	rs376189812	C/G	rs376189812	*TBX3*	12q24.21	exon7:c.G1140C	exon7:p.E380D	12.25
12	115118722	rs35069811	G/A	rs35069811	*TBX3*	12q24.21	exon2:c.C619T	exon2:p.L207L	12.99
12	115120811	chr12:115120811	C/A	chr12:115120811	*TBX3*	12q24.21	exon1:c.G195T	exon1:p.M65I	12.77
**14**	**23598952**	**rs7157021**	**G/A**	**rs7157021**	***SLC7A8***	**14q11.2**	**exon9:c.T1170T**	**exon9:p.Y390Y**	**13.28**
14	23598976	rs17183863	G/A	rs17183863	*SLC7A8*	14q11.2	exon9:c.C1146T	exon9:p.S382S	12.29
**14**	**64694749**	**rs17225885**	**T/G**	**rs17225885**	***ESR2***	**14q23.2**	**exon8:c.A1421C**	**exon8:p.K474T**	**14.43**
14	64724051	rs1256049	C/T	rs1256049	*ESR2*	14q23.2	exon11:c.G984A	exon11:p.V328V	13.06
**14**	**94844843**	**rs1303**	**T/G**	**rs1303**	***SERPINA1***	**14q32.13**	**exon7:c.A1200C**	**exon7:p.E400D**	**16.32**
14	94844975	rs9630	G/A	rs9630	*SERPINA1*	14q32.13	exon7:c.C1068T	exon7:p.A356A	12.33
14	94845824	chr14:94845824	CCT/.	chr14:94845824	*SERPINA1*	14q32.13	exon6:c.1040delA	exon6:p.E347fs	12.24
14	94847285	rs1049800	A/G	rs1049800	*SERPINA1*	14q32.13	exon5:c.T840C	exon5:p.D280D	12.35
14	94847351	rs34112109	C/T	rs34112109	*SERPINA1*	14q32.13	exon5:c.G774A	exon5:p.K258K	12.26
14	94847415	rs6647	G/A	rs6647	*SERPINA1*	14q32.13	exon5:c.T710T	exon5:p.V237V	12.37
14	94849201	rs709932	C/T	rs709932	*SERPINA1*	14q32.13	exon4:c.G374A	exon4:p.R125H	12.45
**15**	**25924539**	**rs1047700**	**T/C**	**rs1047700**	***ATP10A***	**15q12**	**exon21:c.A4449G**	**exon21:p.Q1483Q**	**12.39**

15	25924798	rs9324127	G/A	rs9324127	*ATP10A*	15q12	exon21:c.C4190T	exon21:p.A1397V	12.24
15	25967033	rs28669028	G/A	rs28669028	*ATP10A*	15q12	exon7:c.C1134T	exon7:p.Y378Y	12.24
15	25969041	rs28377484	C/T	rs28377484	*ATP10A*	15q12	exon6:c.G1107A	exon6:p.L369L	12.42
15	25969090	rs17116056	G/T	rs17116056	*ATP10A*	15q12	exon6:c.C1058A	exon6:p.S353Y	13.09
15	25971153	rs116375025	G/A	rs116375025	*ATP10A*	15q12	exon5:c.C924T	exon5:p.C308C	12.44
15	26026292	rs140139603	G/A	rs140139603	*ATP10A*	15q12	exon2:c.C528T	exon2:p.I176I	12.44
**15**	**40698039**	**rs148189323**	**C/T**	**rs148189323**	***IVD***	**15q15.1**	**exon1:c.C20T**	**exon1:p.A7V**	**14.46**
**15**	**48500116**	**rs139471047**	**A/G**	**rs139471047**	***SLC12A1***	**15q21.1**	**exon2:c.A200G**	**exon2:p.Q67R**	**15.34**
15	48522594	chr15:48522594	G/A	chr15:48522594	*SLC12A1*	15q21.1	exon7:c.G869A	exon7:p.S290N	12.48
15	48539129	rs138588696	C/T	rs138588696	*SLC12A1*	15q21.1	exon12:c.C1476T	exon12:p.F492F	12.66
15	48539587	rs6493311	C/T	rs6493311	*SLC12A1*	15q21.1	exon13:c.T1614T	exon13:p.Y538Y	12.50
**15**	**58830656**	**rs113174258**	**G/A**	**rs113174258**	***LIPC***	**15q21.3**	**exon2:c.G213A**	**exon2:p.T71T**	**15.12**
15	58830707	rs7175412	C/T	rs7175412	*LIPC*	15q21.3	exon2:c.C264T	exon2:p.H88H	12.52
15	58860963	rs6074	C/A	rs6074	*LIPC*	15q21.3	exon9:c.C1437A	exon9:p.T479T	12.53
**16**	**1203830**	**rs191613214**	**A/A**	**rs191613214**	***CACNA1H***	**16p13.3**	**exon2:c.G93A**	**exon2:p.E31E**	**18.25**
16	1203851	chr16:1203851	C/A	chr16:1203851	*CACNA1H*	16p13.3	exon2:c.C114A	exon2:p.R38R	12.55
16	1252016	chr16:1252016	TCAC/T	rs760736442	*CACNA1H*	16p13.3	exon9:c.1567_1569del	exon9:p.523_523del	12.26
16	1270871	rs59385968	C/T	rs59385968	*CACNA1H*	16p13.3	exon35:c.C6939T	exon35:p.P2313P	12.57
**16**	**16043641**	**chr16:16043641**	**C/T**	**rs562494702**	***ABCC1***	**16p13.11**	**exon1:c.C33T**	**exon1:p.G11G**	**15.58**
16	16101738	chr16:16101738	C/T	rs745945401	*ABCC1*	16p13.11	exon2:c.C114T	exon2:p.L38L	12.59
16	16218655	rs28363996	C/T	rs28363996	*ABCC1*	16p13.11	exon25:c.C3600T	exon25:p.A1200A	12.26
16	16219729	rs9933640	C/T	rs9933640	*ABCC1*	16p13.11	exon26:c.C3780T	exon26:p.A1260A	12.61
16	16228242	rs2230671	G/A	rs2230671	*ABCC1*	16p13.11	exon28:c.G4002A	exon28:p.S1334S	12.78
16	16230427	rs34526519	C/T	rs34526519	*ABCC1*	16p13.11	exon29:c.C4218T	exon29:p.A1406A	12.65
16	16230442	rs34327330	C/T	rs34327330	*ABCC1*	16p13.11	exon29:c.C4233T	exon29:p.F1411F	12.64

16	16232272	rs36115566	G/A	rs36115566	*ABCC1*	16p13.11	exon30:c.G4344A	exon30:p.T1448T	12.85
16	16232380	rs35148086	C/T	rs35148086	*ABCC1*	16p13.11	exon30:c.C4452T	exon30:p.I1484I	12.66
**16**	**30078564**	**rs368691859**	**A/G**	**rs368691859**	***ALDOA***	**16p11.2**	**exon3:c.A151G**	**exon3:p.T51A**	**16.12**
16	30078907	rs76767223	A/G	rs76767223	*ALDOA*	16p11.2	exon8:c.A249G	exon8:p.T83T	12.68
**17**	**42327874**	**rs45497993**	**A/G**	**rs45497993**	***SLC4A1***	**17q21.31**	**exon20:c.T2688C**	**exon20:p.D896D**	**15.69**
17	42328598	rs5026	C/T	rs5026	*SLC4A1*	17q21.31	exon19:c.G2584A	exon19:p.V862I	12.27
17	42328928	rs139912334	C/T	rs139912334	*SLC4A1*	17q21.31	exon18:c.G2340A	exon18:p.L780L	12.71
17	42332587	rs5020	A/G	rs5020	*SLC4A1*	17q21.31	exon15:c.T1878C	exon15:p.D626D	12.22
17	42334822	rs45568837	C/T	rs45568837	*SLC4A1*	17q21.31	exon13:c.G1522A	exon13:p.E508K	12.73
17	42335443	rs150913170	G/A	rs150913170	*SLC4A1*	17q21.31	exon11:c.C1193T	exon11:p.T398I	12.74
17	42335944	rs5013	C/T	rs5013	*SLC4A1*	17q21.31	exon10:c.G924A	exon10:p.L308L	12.55
17	42338945	rs5036	T/C	rs5036	*SLC4A1*	17q21.31	exon4:c.A166G	exon4:p.K56E	12.76
**19**	**7125297**	**rs1799817**	**G/A**	**rs1799817**	***INSR***	**19p13.2**	**exon17:c.C3255T**	**exon17:p.H1085H**	**18.77**
19	7126632	rs191756282	G/A	rs191756282	*INSR*	19p13.2	exon16:c.C2976T	exon16:p.Y992Y	12.78
19	7132218	rs111502197	C/T	rs111502197	*INSR*	19p13.2	exon14:c.G2793A	exon14:p.A931A	12.49
**19**	**11222300**	**rs11669576**	**G/A**	**rs11669576**	***LDLR***	**19p13.2**	**exon8:c.G1171A**	**exon8:p.A391T**	**16.28**
19	11224265	rs5930	G/A	rs5930	*LDLR*	19p13.2	exon10:c.A1413A	exon10:p.R471R	12.81
19	11240268	chr19:11240268	T/C	chr19:11240268	*LDLR*	19p13.2	exon17:c.T2469C	exon17:p.F823F	12.55
**19**	**34855821**	**chr19:34855821**	**G/A**	**rs549433538**	***GPI***	**19q13.11**	**exon1:c.G7A**	**exon1:p.A3T**	**14.83**
19	34868415	chr19:34868415	G/A	rs867154141	*GPI*	19q13.11	exon6:c.G410A	exon6:p.R137Q	12.84
19	34868642	rs1801015	A/G	rs1801015	*GPI*	19q13.11	exon7:c.A489G	exon7:p.G163G	12.85
19	34872382	rs1864139	G/A	rs1864139	*GPI*	19q13.11	exon10:c.G762A	exon10:p.K254K	12.66
19	34890198	rs34604585	G/C	rs34604585	*GPI*	19q13.11	exon16:c.G1356C	exon16:p.A452A	12.22
**20**	**25259006**	**rs2228976**	**G/T**	**rs2228976**	***PYGB***	**20p11.21**	**exon8:c.G907T**	**exon8:p.A303S**	**18.88**
20	25260931	rs2227890	A/G	rs2227890	*PYGB*	20p11.21	exon10:c.A1122G	exon10:p.A374A	14.39

Supplemental methods

1. Introduction

The African continent harbours the greatest genetic and environmental diversity[1, 2] and has the highest health burden per capita[3], yet there is a scarcity of large-scale disease-specific genome studies of African populations[4, 5]. Sickle cell disease (SCD) has its highest burden in Africa, particularly in Central and West Africa. Here, we report the first study on deleterious variants in deep whole exome sequencing of 192 individuals. This is the first data on whole exome sequencing landscape of SCD in Africa, which has generated a novel database of candidate modifier genes. The exome data provided will serve the global community as it represents the first step of a series of studies on the genomic architecture of SCD, which will be enhanced by the establishment of major NIH-funded research consortia for the study of SCD in Africa[6]. **Figure S1** describes the overall pipeline used for data generation and analyses.

2. Sample Collection

2.1 *Ethics Statement*

The study was performed in accordance with the guidelines of the Helsinki Declaration. Ethical approval was given by the National Ethical Committee Ministry of Public Health, Republic of Cameroon (No 033/CNE/DNM/07) and the University of Cape Town, Faculty of Health Sciences Human Research Ethics Committee (HREC RE: 132/2010). Written and signed informed consent was obtained from the adult participants who were 18 years or older, and for the children consent was obtained from parents/guardians with an assent from the participants older than seven years of age.

2.2 *Patients and assessment of clinical events*

The recruitment for the discovery group was conducted in Cameroon at the Yaoundé Central Hospital and Laquintinie Hospital in Douala, as previously described[7]. Briefly, socio-demographic and clinical data were collected by means of a structured questionnaire. Anthropomorphic variables were

measured in the outpatient setting. Routine blood counts of patients and haemoglobin (Hb) electrophoresis were conducted on arrival at the hospital. In the present study, three sub-groups of SCD patients were included: 1) the "stroke" group made of SCD patients with at least one clinical episode of overt stroke, a devastating complication of SCD, occurring in 11% of patients before age 20 years and considered to be a proxy of severity[8], influenced by genetic modifiers[9]; 2) the "long survivor" SCD group made of patients older than 40 years considered here as the most genetically fit; whose cut off was based on life expectancy of 43 years for SCD-HbSS in the cooperative study conducted four decades ago in the USA8; 3) the "random" group made of SCD patients randomly selected among clinical stable patients without incidence of any cerebrovascular disease by clinical criteria and younger than 40 years.

The replication cohort consisted of adult SCD patients recruited at the Haematology Clinic, Groote Schuur Hospital in Cape Town, that are mostly recent migrants from other sub-Saharan African countries where SCD is prevalent[10]. In order to have a similar genetic background for this replication group, only patients from Demographic Republic of Congo (DRC) were included in the present study. Details of sample information are in **Table S2**

2.3 Control participants

A total of 58 ethnically matched Cameroonian controls were randomly recruited from healthy blood donors in Yaoundé[11] and volunteered their participation in the study. Only individuals, without HbS mutation and that were homozygous HbAA as confirmed by molecular analysis, were included.

3. Library Construction and Sequence

3.1 Molecular methods: Sickle cell anaemia mutation, β-globin gene cluster haplotypes, and 3.7 kb α-globin gene deletion

DNA was extracted from peripheral blood following the manufacturer's instructions (Puregene Blood Kit; Qiagen, Hilden, Germany). Molecular analysis was performed to determine the presence of the sickle mutation and was carried out on 200 ng DNA by polymerase chain reaction (PCR) to amplify a 770 bp segment of the β-globin gene. This was, followed by DdeI restriction analysis of the PCR product[12,13]. Using published primers and

methods, five restriction fragment length polymorphism (RFLP) sites in the β-globin gene cluster were amplified to analyse the XmnI (5'Gγ), HindIII (Gγ), HindIII (Aγ), HincII (3ψβ') and HinfI (5'β) for the HBB haplotype background[11]. The 3.7 kb α-globin gene deletion was successfully screened, using the expand-long template PCR (Roche Diagnostics, Basel, Switzerland), as previously published[11,14].

3.2 Whole Exome Sequencing and Accuracy of exome variant detection

DNA samples underwent sequencing at the Omega Bioservices, Omega Bio-tek, Inc, Emory University USA. DNA concentrations were accurately determined using a picogreen fluorescent detection method (Quant-iT; Invitrogen). Equal amounts of DNA from each sample constituting a pool were manually combined before WES. The Roche Nimblegen SeqCap EZ MedExome v2.0 (~47Mb target) were used for sequence capture. The MedExome Kit was designed to provide sequencing coverage for gene annotations from several sources (i.e. Refseq, Ensemble and GENCODE) as well as miRBase, and enhanced coverage of medically relevant genes such as those in GeneTests, OMIM, ClinVar. All protocols for shotgun library construction for exome capture have been automated on a Perkin-Elmer Janus II liquid handling robot or multi-channel pipettors. After shotgun library construction, library concentrations and molecular weight distributions are determined in parallel on an Agilent Bioanalyzer in order to flag low-quality libraries prior to exome capture. Samples passing quality control, library preparation and exome capture are sequenced on an Illumina HiSeq 4000 sequencer. WES was generated on the HiSeq X.

4. Reads Mapping and Alignment

To insure the forward and reverse reads are of high quality and appropriate length, we evaluate these using used both FastQC[15] and SolexaQA++[16]. From the UCSC database[17], we obtained the human reference genome, version hg19 (build37), together with its gene annotation. The reads were aligned to the UCSC hg19 (build37) complete reference genome using BWA[18,19]. Using the Picard tool kit[20], the duplicate reads were marked, and after alignment, the BAM files were sorted by coordinates, indexed and read groups were added via Picard[20]. BAM files were re-ordered according to UCSC hg19[17]. Insertions and deletions at the end of the reads can misguide the aligners into mis-aligning with mismatches. This artificial mismatch can

mislead base quality score recalibration and variant detection. To address this issue, we used the Genome Analysis Toolkit (GATK) software[20] for local realignment along all reads at a problematic locus to create a cleaned version of the BAM file and found a best consensus sequence that, together with the reference, best fits the reads in a pile. We used the 1000 genomes phase 1 INDELs and Mills and 1000 genomes gold standard INDELs[21] to drive the process. Using these known sites improves the accuracy. After INDEL realignment we applied Picard "FixMateInformation" to recalculate read pair information to see if it has changed. At this stage all 192 samples have > 79% of target bases covered to ~35x.

5. Variant Calling

As different calling methods produce a large number of differing variants and previous studies have demonstrated that these methods have differing advantages[22,23,24], we adopted an ensemble approach implemented in VariantMetaCaller[25] in each data set of subjects group and the all dataset of 192 subjects. To detect SNPs and short indels, we combined information generated from three independent variant caller pipelines (**Figure S1**): (1) An incremental joint variant discovery implemented in GATK 3.0 HaplotypeCaller[20], which calls samples independently to produce gVCF files and leverages the information from the independent gVCF file to produce a final call-set at the genotyping step; (2) FreeBayes[26] and (3) samtools via mpileup[27] variant callers (**Figure S1**). The best practice specific to each caller were adopted[28].

6. Variant Calling Quality Control and Final Call-set

Before applying the ensemble approach from the resulting variant sets per subject group and all subjects from each these three callers respectively, we filtered each resulting VCF files using the GATK tool "VariantFiltration".

6.1 Flagging Variants

We added additional filter levels to each call set as follows: (1) If 3 SNPs are detected within a window of 10 base-pairs, the site will be flagged as a "SNPCluster" in the FILTER column (2) if 4 or more alignments having a mapping quality of MQ = 0 (which means it maps to different locations equally well) and the number of alignments that mapped ambiguously are more than a tenth of all alignments, it is difficult to decipher artefacts and true

differences. These sites will be flagged as “HARD_TO_VALIDATE”, (3) SNPs which are covered by less than 5 reads may be potential artefacts and these sites was flagged as “LowCoverage”, (4) SNPs having a SNP quality below 30 are typically artefacts, were flagged as “VeryLowQual”, (5) SNPs having a quality score between 30 and 50 are potential artefacts, flagged as “LowQual”, (6) SNPS having a QD score < 1.5 are indicative of false positive calls and artefacts, flagged as “LowQD” (7) and SNPs covered only by sequences on the same strand are often artefacts, was flagged as “StrandBias”.

6.2 Variants Quality Control Assessment Prior Final Call-set

Variants flagged “VeryLowQual”, “LowQual”, “LowQD” and “StrandBias” were removed in each VCF files. Exomes VCF files were assessed according to the total reads, coverage distribution, raw error rates, transition/transversion (Ti/Tv) ratios (3.2), comparison of genomic sex to recorded sex, distribution of known variants (relative to dbSNP), CytoSNP array fingerprint concordance > 99%, homozygosity, heterozygosity, and sample contamination validation. Additionally, variant sites that strongly deviate from Hardy-Weinberg equilibrium (p-value < 5x 10-5) were flagged. These criteria reduce the inclusion of false-positive variant calls during the ensemble of the VCF files.

The final call-set from each subjects group, were produced from VariantMetaCaller[25], a support vector machines approach that combined the hard-filtered VCF files obtained from these three variants callers.

7. Variant Annotation and Mutation Prioritization

After high confidence variants were called using VariantMetaCaller from each dataset include 3 Cameroonian SCD categories; namely 56 moderate SCD patients, 26 stroke SCD patients, 23 survival SCD individuals for the discovery analysis, 58 Cameroon control samples and 29 SCD patients from the DRC for [replication] analysis. We used ANNOVAR[29] to independently perform gene-based annotation in each final VCF data set to determine whether SNPs cause protein coding changes and produce a list of the amino acids that are affected. We used ANNOVAR “2016Dec18” setting, where the population frequency, pathogenicity for each variant was obtained from 1000 Genomes exome[21], Exome Aggregation Consortium[30] (ExAC), targeted exon datasets and COSMIC[31]. Gene functions were obtained from RefGene[32] and different functional predictions were obtained from ANNOVAR's

library, which contains up to 21 different functional scores including SIFT[33,34], LRT[35], MutationTaster[36], MutationAssessor[37,38], FATHMM[39], fathmm-MKL[39], RadialSVM[40], LR[40], PROVEAN[40], MetaSVM[40], MetaLR[40], CADD[41], GERP++[42], DANN[29], M-CAP[29], Eigen[29], GenoCanyon[29], Polyphen2 HVAR[43], Polyphen2 HDIV[43], PhyloP[44] and SiPhy[44]. We additionally included conservative and segmental duplication sites, dbSNP code and clinical relevance reported in dbSNP[45]. From each resulting functional annotated data set, we independently filtered for predicted functional status (of which each predicted functional status is of "deleterious" (D), "probably damaging" (D), "disease_causing_automatic" (A) or "disease_causing" (D).[46,47,49]) from SIFT, LRT, MutationTaster, MutationAssessor, FATHMM, fathmm-MKL, RadialSVM, LR, PROVEAN, MetaSVM, MetaLR, CADD, GERP++, DANN, M-CAP, Eigen, GenoCanyon, Polyphen2 HVAR, Polyphen2 HDIV, PhyloP, and SiPhy.

We used a casting vote approach, by retaining only a variant if it had at least 17 predicted functional status “D” or “A“out of 21. Second, the retained variants from each data set were further filtered for rarity, exonic variants, and nonsynonymous mutations and with high quality call as describe above, yielding a final candidate list of predicted mutant variants in each subject group, including the replication group. To compare the results from the above strategy, we re-applied FATHMM[22], a disease-specific weighting scheme, which uses a Hidden Markov Models prediction algorithm capable of discriminating between disease-causing mutations and neutral polymorphisms. We report on the aggregated SiPhy score from all identified mutants SNPs within gene.

8. Network and Enrichment Analysis

8.1 Reconstruction of Sub-network

To identify sub-networks of interactive mutant variant genes (**section 7** above) or from gene-specific difference in SNPs frequencies genes (**section 13** below) from each SCD category occurred, we used a comprehensive human Protein-Protein Interaction (PPI) network[50,51,52,53,54,55] to analyse how each set of variant genes are layered and interact within a biological network, thus extracting a sub-network. A clustering script in R’s (R Core Team, 2016)

igraph package was used to determine a network plot that would allow us to identify the hub proteins in the sub-networks.

8.2 Enrichment of genes within Sub-network

We examined these candidate variants genes from either mutation prioritization (**section 7** above) or gene-specific difference in SNPs frequencies (see **section 13** below) are interact with other genes and how in the formed sub-network they can be associated with human phenotypes and what are their pathways, biological processes and molecular functions. To address this, we used a custom script and adopted to compare our results with different tools including DAVID[56] and PANTHER[57]. We additionally conducted an enrichment analysis using the Enrichr software[58]. The most significant pathway enriched for genes in the networks were selected from KEGG[59], Panther[57], Biocarta[60] and Reactome[61], and gene ontologies from the Gene Ontology Consortium[62] were defined for cellular component, biological process and molecular function.

9. Further Characterization of Enriched Sub-networks.

To identify the association between each sub-network Sj, (j=1,…,T) within n_1,..., n_T genes to human pathway, $P_k \in P$the set of human pathways. We obtained 1,547 annotated pathways[51,52,53,54,55] and collected more than 107 annotated pathways from the KEGG[59], BioCarta[60] and Ambion GeneAssistTM Pathway Atlas pathway databases. We downloaded genomic co-ordinates for all genes from the NCBI ftp-server[45] and retained only entries for the human reference sequence. We assigned the SNPs located within a gene or in LD within less than 40kb distance up/downstream of the gene using dbsnp database[45]. Let a number of genes in the intersection between genes within Sj and genes within pathwayP_k. Let b be the number of genes in the intersection between genes within Sj and those in the union of all pathways$P_{k(k=1,...,K)}$. Let n be the number of genes in the intersection between genes in the P_j pathway and those in the union of all pathways $P_{k(k=1,...,K)}$with$k \neq j$, and m be the total number of genes in all pathways $P_{k(k=1,...,K)}$. We computed the statistic of significance of overlap between sub-network S_j, of n_t genes and a given pathway P_k using the z-score (ZS), which employs the binomial proportion test,

$$Z_S = \frac{\left(\frac{a}{n} - \frac{b}{m}\right)}{\sqrt{\frac{1-\frac{b}{m}}{m}}}$$

10. Procedure to Aggregating SNPs Summary Statistics at the Gene level.

From each subject group, we estimated the statistical significance at gene level from the list of resulting genes associated to predict mutant variants (**section 7** above) or from the list of candidate gene-specific difference in SNP frequencies (**section 13** below). In doing so, we aggregated the P-values from SNPs 40kb downstream and upstream gene region (exon) as per gene-based annotation file in our exome datasets. Under the null hypothesis, p-values p_k (k = 1,...,L) for a test-statistic with a continuous distribution are uniformly distributed in the interval [0,1]. It follows that a parametric cumulative distribution function F can be chosen and p_k can be transformed into quantiles according to $q_k = F^{-1}(p_k)$. The combined test statistic $C^p = \frac{\sum_{k=1}^{L} q_k}{\sqrt{L}}$ is a sum of independent and identically distributed random variables q_k. To account for the independence assumption given correlation among neighbouring genomic markers, we implement the Stouffer-Liptak method accounting for spatial correlations among SNPs within a gene or SNPs within a given sub-network. The overall statistic can be obtained by $p = \Phi(C^p)$, in which Φ is the cumulative distribution function of the standard normal distribution. We apply the Benjamini-Hochberg false-discovery correction method to control the type I error rate and account for gene/sub-network differences in the numbers of associated SNPs. From each subject group, we reported on the overall statistical significance of gene mutational burden gene, p-values after the Benjamini-Hochberg false-discovery correction.

11. Phased and Haplotypes Inference

From the VCF file resulted from joint variant calling (section VI above), a merged dataset of all 192 samples (58 Hb AA Cameroon controls, a replication cohort of 29 SCD patients from DRC, 56 random, 26 Stroke, and 23 survival SCD patients and 58 controls from Cameroon. We further conducted quality control to remove all structured, indel, multi-allelic variants and those with low minor allele frequency (MAF < 0.05) prior to phasing. We first phased and inferred the haplotypes using Eagle[66] from the resulting curated data.

Second, we extracted and re-phased samples from the 1000 Genomes Project[21]. To merge our exomic haplotypes to those from 1000 Genomes, we computed a cross-imputation using impute2[67]. We performed further quality control after the imputation, which consisted of removal of variants with low minor allele frequency (MAF < 0.05); low genotypes call (<= 95%) and imputation accuracy (< 955) prior to re-phasing using Eagle66. We performed a post-phasing quality control in which we checked the switch-error between our exomic haplotypes panel and the exomic haplotypes merged to 1000 Genomes panel[21], where 99.7% and 97,05% of the sites were with no phase switch-error in both panels, respectively. We further compared sites discordance between these haplotype panels and independently with their original VCF file prior phasing. The only site with phase switch-errors showed discrepancies in MAF and were therefore removed.

12. Fine-scale Population Structure

Population structure analyses were performed to characterize the genetic contributions to our 192 patient samples. We first tested whether populations conform to an isolation-by-distance model or whether there is strong heterogeneity among populations relative to geographic distance. To this end, we have merged our 192 samples with data from 1000 Genomes Projects in which we performed principal component analysis using smartpca in the EIGENSOFT package[68,69] and included all SNP haplotypes shared between the populations analysed. Population-specific pair-wise genetic distance (F_ST) and a phylogeny tree was computed from smartpca. All the 192 samples, including the SCD from DRC were observed to cluster together,

indicating geographic and genetics proximity. Table below displays the population-specific F_ST, indicating close genetic relatedness between SCD groups from Cameroon as well as relatedness between SCD patients from DRC and the Control Cameroon group. The data suggests an isolation-by-distance model.

Table S3 Pair-wide Population genetic distance among 58 Hb AA Cameroon controls, a replication cohort of 29 SCD patients from DRC, 56 random, 26 Stroke and 23 survivor SCD patients from Cameroon.

FST	SCD Cameroon Long Survivor	SCD Cameroon Random	SCD DRC	SCD Cameroon Stroke	Control Cameroon
SCD Cameroon Long Survivor	-	0·001	0·006	0·002	0·003
SCD Cameroon Random	0·001	-	0·004	0·001	0·002
SCD DRC	0·006	0·004	-	0·005	0·003
SCD Cameroon Stroke	0·002	0·001	0·005	-	0·002
Control Cameroon	0·003	0·002	0·003	0·002	-

13. Unusual Genetic Difference: SNP-specific differences in allele frequency

Similarly, to[70,71], and assuming the population evolved under the Wright-Fisher model, selective neutrality and with an expected number of mutations, we used a step-wise constant effective population size[70] to compute allele frequency spectrum. We secondly computed the pair-wise group frequency

spectrum difference at SNP level, thus SNP allele frequency spectrum difference are assigned to a given gene if they are located within the gene's 40kb downstream or upstream using dbsnp database, thus aggregating SNPs allele frequency difference into genes[51,52,53,54,55]. To this end, let $f_i^1$and f_i^2 be the allele frequency spectrum in group 1 and group 2, respectively. To minimize deviation from the normality assumption, SNPs with minor allele frequencies < 0.05 are excluded. Thus, at a given locus i, the difference $f_i = (f_i^1 - f_i^2)$between observed variant allele frequencies of two groups 1 and 2. Let gene j (j = 1,.., N) has n_j associated SNPs, thus $d_j = \frac{\sum_{i=1}^{n_j} f_i}{n_j}$ is gene j (j=1,..,N) frequency difference from group 1 and 2, and can be approximated as a normal distribution under neutral drift with mean 0 and variance[71,72]

$$d(1-d)\left(2\mathrm{F}_{\mathrm{ST}} + \frac{1}{n_j}\right)$$

where F_{ST} is the genetic distance between the group 1 and 2

$$d = \frac{\sum_{i=1}^{n_j}(f_i^1 + f_i^2)}{2}$$

total variant allele counts in each population, and p is the overall gene-specific ancestral frequency that is approximated as the average of the two observed variant allele frequencies from n_jSNPs associated to gene j. Similar to[70,71], we test unusual gene-specific difference U_{12} from groups 1 and 2 as follows

$$U_{12}^j = \frac{(d_j)^2}{d(1-d)\left(2\mathrm{F}_{\mathrm{ST}} + \frac{1}{n_j}\right)}$$

This equation is χ^2 distributed with 1 degree of freedom (d.o.f). An excess of large values of the χ^2 statistic indicates deviations from the null model, suggesting the action of natural selection[71,72]. We applied this method to each group-pair from the three groups of SCD. Finally, SNP-specific unusual allele frequency summary statistics of SNPs within gene region can be aggregated (**see section** 10 above) to obtain gene-specific differences in SNP frequencies.

16. Supplemental references

1. Tishkoff, S. A. et al. The genetic structure and history of Africans and African Americans. Science 324, 1035-44 (2009).

2. Henn, B. M. et al. Distance from sub-Saharan Africa predicts mutational load in diverse human genomes. Proc. Natl. Acad. Sci. 113, E440-E449 (2016).

3. Ramsay, M., Tiemessen, C. T., Choudhury, A. & Soodyall, H. Africa: the next frontier for human disease gene discovery? Hum. Mol. Genet. 20, R214-20 (2011).

4. Bustamante, C. D., Burchard, E. G. & De la Vega, F. M. Genomics for the world. Nature 475, 163-5 (2011).

5. H3Africa Consortium et al. Research capacity. Enabling the genomic revolution in Africa. Science 344, 1346-8 (2014).

6. Makani, J., Ofori-Acquah, S.F., Tluway, F., Mulder, N. and Wonkam, A., 2017. Sickle cell disease: tipping the balance of genomic research to catalyse discoveries in Africa. Lancet (London, England), 389(10087), p.2355.

7. Wonkam, A., Bitoungui, V.J.N., Vorster, A.A., Ramesar, R., Cooper, R.S., Tayo, B., Lettre, G. and Ngogang, J., 2014. Association of variants at BCL11A and HBS1L-MYB with hemoglobin F and hospitalization rates among sickle cell patients in Cameroon. PloS one, 9(3), p.e92506.

8. Platt, O.S. et al. Mortality in sickle cell disease life expectancy and risk factors for early death. New England Journal of Medicine. 330(23), 1639-1644 (1994).

9. Flanagan JM, Sheehan V, Linder H, et al. (2013). Genetic mapping and exome sequencing identify 2 mutations associated with stroke protection in pediatric patients with sickle cell anemia. Blood 121, 3237-3245.

10. Pule, G.D., Ngo Bitoungui, V.J., Chetcha Chemegni, B., Kengne, A.P. and Wonkam, A., 2016. Studies of novel variants associated with Hb F in Sardinians and Tanzanians in sickle cell disease patients from Cameroon. Hemoglobin, 40(6), pp.377-380.

11. Rumaney, M.B., Bitoungui, V.J.N., Vorster, A.A., Ramesar, R., Kengne, A.P., Ngogang, J. and Wonkam, A., 2014. The co-inheritance of alpha-thalassemia and sickle cell anemia is associated with better hematological indices and lower consultations rate in Cameroonian patients and could improve their survival. PLoS One, 9(6), p.e100516.

12. Saiki RK, et al. Enzymatic amplification of beta-globin genomic sequences and restriction site analysis for diagnosis of sickle cell anemia. Science (New York, N.Y.) 1985; 230:1350-1354.

13. Darbari, D.S., Wang, Z., Kwak, M., Hildesheim, M., Nichols, J., Allen, D., Seamon, C., Peters-Lawrence, M., Conrey, A., Hall, M.K. and Kato, G.J., 2013. Severe painful vaso-occlusive crises and mortality in a contemporary adult sickle cell anemia cohort study. PLoS One, 8(11), p.e79923.

14. Bitoungui, V.J.N., Pule, G.D., Hanchard, N.,Ngogang, J. & Wonkam, A. (2015) Beta-globin gene haplotypes among cameroonians and review of the global distribution: is there a case for a single sickle mutation origin in Africa? Omics: a Journal of Integrative Biology, 19, 171-179.

15. Auwera, G.A. et al. From FastQ data to high-confidence variant calls: the genome analysis toolkit best practices pipeline. Current Protocols in Bioinformatics, 11-10 (2013).

16. Cox, M.P.et al. SolexaQA: At-a-glance quality assessment of Illumina second-generation sequencing data. BMC bioinformatics, 11(1), p.1 (2010).

17. Rosenbloom, K.R. et al. The UCSC Genome Browser database: 2015 update. Nucleic Acids Res. 43(Database issue):D670-81 (2015).

18. Li, H. et al. Fast and accurate short read alignment with Burrows-Wheeler transform. Bioinformatics, 25(14):1754-1760 (2009).

19. Li H, Ruan J, Durbin R: Mapping short DNA sequencing reads and calling variants using mapping quality scores. Genome Res, 18(11):1851-1858 (2008).

20. McKenna, A. et al. The Genome Analysis Toolkit: a MapReduce framework for analyzing next-generation DNA sequencing data. Genome Res. 20(9):1297-1303 (2010).

21. 1000 Genomes Project Consortium, 2012. An integrated map of genetic variation from 1,092 human genomes. Nature, 491(7422), pp.56-65.

22. DePristo, M.A. et al. A framework for variation discovery and genotyping using next-generation DNA sequencing data. Nat Genet, 43(5):491-498 (2011).

23. Liu, X. et al. Variant callers for next-generation sequencing data: a comparison study. PLoS One. 8(9):e75619 (2013).

24. Liu, Q. et al. Steps to ensure accuracy in genotype and SNP calling from Illumina sequencing data. BMC Genomics 13 Suppl 8:S8 (2012).

25. Gézsi, A. et al. VariantMetaCaller: automated fusion of variant calling pipelines for quantitative, precision-based filtering. BMC genomics, 16(1), p.1 (2015).

26. Li, H. A. et al. statistical framework for SNP calling, mutation discovery, association mapping and population genetical parameter estimation from sequencing data. Bioinformatics. 27(21):2987-93 (2011).

27. Garrison, E. et al. Haplotype-based variant detection from short-read sequencing. arXiv preprint arXiv:1207.3907 (2012).

28. Cornish, A. et al. A comparison of variant calling pipelines using genome in a bottle as a reference. BioMed research international, 456479, 11 (2015).

29. Wang, K. el at. ANNOVAR: Functional annotation of genetic variants from next-generation sequencing data Nucleic Acids Research, 38:e164 (2010).

30. Karczewski, K.J., Weisburd, B., Thomas, B., Solomonson, M., Ruderfer, D.M., Kavanagh, D., Hamamsy, T., Lek, M., Samocha, K.E., Cummings, B.B. and Birnbaum, D., 2016. The ExAC browser: displaying reference data information from over 60 000 exomes. Nucleic acids research, 45(D1), pp.D840-D845.

31. Forbes, S.A., Beare, D., Gunasekaran, P., Leung, K., Bindal, N., Boutselakis, H., Ding, M., Bamford, S., Cole, C., Ward, S., Kok, C.Y., Jia, M., De, T., Teague, J.W., Stratton, M.R., McDermott, U., Campbell, P.J., 2015. COSMIC: Exploring the world's knowledge of somatic mutations in human cancer. Nucleic Acids Res. 43, D805-D811.

32. O'Neill, K., Pujar, S., Rangwala, S.H., Rausch, D., Riddick, L.D., Schoch, C., Shkeda, A., Storz, S.S., Sun, H., Thibaud-Nissen, F., Tolstoy, I., Tully, R.E., Vatsan, A.R., Wallin, C., Webb, D., Wu, W., Landrum, M.J., Kimchi, A., Tatusova, T., DiCuccio, M., Kitts, P., Murphy, T.D., Pruitt, K.D., 2016. Reference sequence (RefSeq) database at NCBI: Current status, taxonomic expansion, and functional annotation. Nucleic Acids Res. 44, D733-D745.

33. Ng, P.C., Henikoff, S., 2003. SIFT: Predicting amino acid changes that affect protein function. Nucleic Acids Res. 31, 3812-3814.

34. Ng, P.C., Henikoff, S., 2006. Predicting the effects of amino acid substitutions on protein function. Annu. Rev. Genomics Hum. Genet. 7, 61-80.

35. Fujita, A., Kojima, K., Patriota, A.G., Sato, J.R., Severino, P., Miyano, S., Dopazo, J., 2011. A fast and robust statistical test based on likelihood ratio with Bartlett correction to identify Granger causality between gene sets. Bioinformatics 27, 2349-2351.

36. Lubeck, E., Coskun, A.F., Zhiyentayev, T., Ahmad, M., Cai, L., 2012. MutationTaster2: mutation prediction for the deep-sequencing age. Nat. Methods Nat. Methods Nat. Methods 9, 743-748.

37. Reva, B., Antipin, Y., Sander, C., 2007. Determinants of protein function revealed by combinatorial entropy optimization. Genome Biol. 8, R232.

38. Reva, B., Antipin, Y., Sander, C., 2011. Predicting the functional impact of protein mutations: Application to cancer genomics. Nucleic Acids Res. 39, 37-43.

39. Shihab, H.A., Gough, J., Mort, M., Cooper, D.N., Day, I.N.M., Gaunt, T.R., 2014. Ranking non-synonymous single nucleotide polymorphisms based on disease concepts. Hum. Genomics 8, 11.

40. Dong, C., Wei, P., Jian, X., Gibbs, R., Boerwinkle, E., Wang, K., Liu, X., 2015. Comparison and integration of deleteriousness prediction methods for nonsynonymous SNVs in whole exome sequencing studies. Hum. Mol. Genet. 24, 2125-2137.

41. Kircher, M., 2014. A general framework for estimating the relative pathogenicity of human genetic variants. Nat. g 46, 310-315.

42. Cooper, G.M., Stone, E.A., Asimenos, G., Green, E.D., Batzoglou, S., Sidow, A., 2005. Distribution and intensity of constraint in mammalian genomic sequence. Genome Res. 15, 901-913.

43. Adzhubei, I.A., Schmidt, S., Peshkin, L., Ramensky, V.E., Gerasimova, A., Bork, P., Kondrashov, A.S., Sunyaev, S.R., 2010. A method and server for predicting damaging missense mutations. Nat. Methods 7, 248-249.

44. Garber, M., Guttman, M., Clamp, M., Zody, M.C., Friedman, N., Xie, X., 2009. Identifying novel constrained elements by exploiting biased substitution patterns. Bioinformatics 25, 54-62.

45. Sherry, S., Ward, M., Kholodov, M., Baker, J., Phan, L., Smigielski, E., Sirotkin, K., 2001. dbSNP: the NCBI database of genetic variation. Nucleic Acids Res 29, 308-11.

46. Wheeler, D.L. et al. Database resources of the National Center for Biotechnology Information. Nucleic Acids Res. 35 (Database issue): D5-1

(2007).

47. Li, M.X. et al. A comprehensive framework for prioritizing variants in exome sequencing studies of Mendelian diseases. Nucleic acids research, p.gkr1257 (2012).

48. Li, M.X. et al. Predicting mendelian disease-causing non-synonymous single nucleotide variants in exome sequencing studies. PLoS Genet, 9(1), p.e1003143 (2013).

49. Sifrim, A.et al. eXtasy: variant prioritization by genomic data fusion. Nature methods, 10(11), 1083-1084 (2013).

50. Robinson, P.N. et al. The human phenotype ontology. Clinical genetics, 77(6), 525-534 (2010).

51. Wang, Q., Yu, H., Zhao, Z. and Jia, P., 2015. EW_dmGWAS: edge-weighted dense module search for genome-wide association studies and gene expression profiles. Bioinformatics, p.btv150.

52. Laoutidis, Z.G. and Luckhaus, C., 2015. The Liptak-Stouffer test for meta-analyses. Biological psychiatry, 77(1), pp.e1-e2.

53. Wu, J. et al. Integrated network analysis platform for protein-protein interactions. Nat Meth 6, 75-77 (2009).

54. Chimusa, E. R. et al. ancGWAS: a Post Genome-wide Association Study Method for Interaction, Pathway, and Ancestry Analysis in Homogeneous and Admixed Populations. Bioinformatics, btv619 (2015).

55. Huang, D.W., Sherman, B.T. and Lempicki, R.A., 2009. Bioinformatics enrichment tools: paths toward the comprehensive functional analysis of large gene lists. Nucleic acids research, 37(1), pp.1-13.

56. Dennis, G., Sherman, B.T., Hosack, D.A., Yang, J., Gao, W., Lane, H.C. and Lempicki, R.A., 2003. DAVID: database for annotation, visualization, and integrated discovery. Genome biology, 4(9), p.R60.

57. Mi, H., Huang, X., Muruganujan, A., Tang, H., Mills, C., Kang, D. and Thomas, P.D., 2016. PANTHER version 11: expanded annotation data from Gene Ontology and Reactome pathways, and data analysis tool enhancements. Nucleic Acids Research, p.gkw1138.

58. Kuleshov, M.V., Jones, M.R., Rouillard, A.D., Fernandez, N.F., Duan, Q., Wang, Z., Koplev, S., Jenkins, S.L., Jagodnik, K.M., Lachmann, A.

and McDermott, M.G., 2016. Enrichr: a comprehensive gene set enrichment analysis web server 2016 update. Nucleic acids research, p.gkw377.

59. Kanehisa, M. and Goto, S., 2000. KEGG: kyoto encyclopedia of genes and genomes. Nucleic acids research, 28(1), pp.27-30.

60. Nishimura, D., 2001. BioCarta. Biotech Software & Internet Report: The Computer Software Journal for Scient, 2(3), pp.117-120.

61. Croft, D., Mundo, A.F., Haw, R., Milacic, M., Weiser, J., Wu, G., Caudy, M., Garapati, P., Gillespie, M., Kamdar, M.R. and Jassal, B., 2013. The Reactome pathway knowledgebase. Nucleic acids research, 42(D1), pp.D472-D477.

62. Reference Genome Group of the Gene Ontology Consortium, 2009. The Gene Ontology's Reference Genome Project: a unified framework for functional annotation across species.PLoS computational biology,5(7), p.e1000431.

63. Evangelou, E. and Ioannidis, J.P., 2013. Meta-analysis methods for genome-wide association studies and beyond. Nature Reviews Genetics, 14(6), pp.379-389.

64. Sul, J.H., Han, B., Ye, C., Choi, T. and Eskin, E., 2013. Effectively identifying eQTLs from multiple tissues by combining mixed model and meta-analytic approaches. PLoS Genet, 9(6), p.e1003491.

65. Benjamini, Y., Drai, D., Elmer, G., Kafkafi, N. and Golani, I., 2001. Controlling the false discovery rate in behavior genetics research. Behavioural brain research, 125(1), pp.279-284.

66. Loh, P.R., Danecek, P., Palamara, P.F., Fuchsberger, C., Reshef, Y.A., Finucane, H.K., Schoenherr, S., Forer, L., McCarthy, S., Abecasis, G.R. and Durbin, R., 2016. Reference-based phasing using the Haplotype Reference Consortium panel. Nature genetics, 48(11), pp.1443-1448.

67. B. Howie, C. Fuchsberger, M. Stephens, J. Marchini, and G. R. Abecasis (2012) Fast and accurate genotype imputation in genome-wide association studies through pre-phasing. Nature Genetics 44(8): 955-959.

68. Patterson, N., Price, A.L. and Reich, D., 2006. Population structure and eigenanalysis. PLoS genetics, 2(12), p.e190.

69. Price, A.L., Patterson, N.J., Plenge, R.M., Weinblatt, M.E., Shadick, N.A. and Reich, D., 2006. Principal components analysis corrects for stratification in genome-wide association studies. Nature genetics, 38(8), pp.904-909.

70. Marth, G.T., Czabarka, E., Murvai, J. and Sherry, S.T., 2004. The allele frequency spectrum in genome-wide human variation data reveals signals of differential demographic history in three large world populations. Genetics, 166(1), pp.351-372.

71. Price, A.L., Helgason, A., Palsson, S., Stefansson, H., Clair, D.S., Andreassen, O.A., Reich, D., Kong, A. and Stefansson, K., 2009. The impact of divergence time on the nature of population structure: an example from Iceland. PLoS Genet, 5(6), p.e1000505.

72. Bhatia, G., Patterson, N., Pasaniuc, B., Zaitlen, N., Genovese, G., Pollack, S., Mallick, S., Myers, S., Tandon, A., Spencer, C. and Palmer, C.D., 2011. Genome-wide comparison of African-ancestry populations from CARe and other cohorts reveals signals of natural selection. The American Journal of Human Genetics, 89(3), pp.368-381.
